# Exosome proteomes reveal glycolysis-related enzyme enrichment in primary canine mammary gland tumor compared to metastases

**DOI:** 10.1186/s12953-023-00226-5

**Published:** 2024-02-28

**Authors:** Hui-Su Kim, Je-Yoel Cho

**Affiliations:** 1 Department of Biochemistry, College of Veterinary Medicine, Research Institute for Veterinary Science, and BK21 FOUR Future Veterinary Medicine Leading Education and Research Center, Seoul National University, Gwanak-ro1, Gwanak-Gu, Seoul, 08826 Republic of Korea; 2 Comparative Medicine Disease Research Center (CDRC), Science Research Center (SRC), Seoul National University, Seoul, 08826 Republic of Korea

**Keywords:** Cancer, Primary tumor, Metastases, Exosome, Proteomics

## Abstract

**Objective:**

Numerous evidence has highlighted the differences between primary tumors and metastases. Nonetheless, the differences in exosomal proteins derived from primary tumor and metastases remain elusive. Here, we aimed to identify differentially expressed exosomal proteins from primary canine mammary gland tumor and metastases to understand how they shape their own tumor microenvironment.

**Methods:**

We clearly distinguished primary canine mammary gland tumors (CHMp) from metastases (CHMm) and profiled the proteins within their secreted exosomes using LC–MS/MS. Moreover, the abundance of glycolysis enzymes (GPI, LDHA) in CHMp exosome was verified with Western blotting, To broaden the scope, we extended to human colorectal cancer-derived exosomes (SW480 vs. SW620) for comparison.

**Results:**

We identified significant differences in 87 and 65 proteins derived from CHMp and CHMm, respectively. Notably, glycolysis enzymes (GPI, LDHA, LDHB, TPI1, and ALDOA) showed specific enrichment in exosomes from the primary tumor.

**Conclusion:**

We observed significant differences in the cellular proteome between primary tumors and metastases, and intriguingly, we identified a parallel heterogeneity the protein composition of exosomes. Specifically, we reported that glycolysis enzymes were significantly enriched in CHMp exosomes compared to CHMm exosomes. We further demonstrated that this quantitative difference in glycolysis enzymes persisted across primary and metastases, extending to human colorectal cancer-derived exosomes (SW480 vs. SW620). Our findings of the specific enrichment of glycolysis enzymes in primary tumor-derived exosomes contribute to a better understanding of tumor microenvironment modulation and heterogeneity between primary tumors and metastases.

**Supplementary Information:**

The online version contains supplementary material available at 10.1186/s12953-023-00226-5.

## Background

Ninety percent of cancer-related deaths are attributed to metastases [1]. To address this high mortality rate, understanding the distinctions between primary tumor and metastases is a crucial task in unravelling the intricacies of disease progression. The primary tumor is where cancer originates and initially manifests, representing the early genetic changes and molecular characteristics that determine its origin [2]. However, an intriguing aspect is that, even if they share the same origin, primary tumors and metastases have distinct cellular characteristics [3]. Based on these disparities, primary tumors and metastases have different communication systems in forming their tumor microenvironment. Accumulating evidence revealed that cancer cells not only directly interact with surrounding cells such as fibroblasts, endothelial cells, and immune cells within the tumor microenvironment but also indirectly create their desired niche by secreting soluble factors [4, 5]. The communication between cancer cells and the surrounding microenvironment is complex and involves various signaling pathways and interactions [6–8]. Through this intricate interplay, cancer cells can modulate the tumor microenvironment to support their survival, growth, invasion, and the formation of metastases [9]. Recently, the role of signaling molecules in regulating the tumor niche has been well established not only for soluble proteins but also for exosomes [10, 11].

Exosomes, a specific type of extracellular vesicle, are secreted by various cells and typically range in size from 30 to 150 nm are found in biological fluids [12]. It is known that cancer cells secrete a higher number of exosomes compared to normal cells. These exosomes contain proteins, RNAs, DNAs, and non-coding RNAs [13, 14]. The components within exosomes are shuttled through various mechanisms within cells, reflecting the characteristics of the parent cells [15]. The composition of exosomes can vary depending on the cell type and the conditions under which they are secreted [16]. Numerous studies have shown that exosomes derived from cancer cells carry oncogenic proteins, mRNAs, and ncRNAs, which can contribute to the formation of a metastatic niche in nearby or distant cells [17–19]. However, many studies tend to overlook the distinct heterogeneity between primary tumors and metastases and treat them without clear distinction.

In this study, our objective was to conduct a detailed profiling and identification of distinct proteins present in exosomes derived from primary tumors compared to those originating from metastases. We used canine mammary gland tumor patients-derived cell lines; CHMp for primary tumor and CHMm for metastases. Through the utilization of LC–MS/MS, we conducted an extensive analysis to uncover unique protein signatures inherent to exosomes derived from primary tumors and metastases. Proteome analysis of these exosomes revealed significant differences between them. More importantly, glycolysis enzymes (GPI, LDHA, TPI1, and ALDOA) were significantly enriched in the exosomes of primary tumors of both canine mammary tumor and human colorectal tumor, compared to metastases.

## Methods

### Cell culture

Canine mammary tumor cells (CHMp and CHMm) were established and obtained from the N. Sasaki lab [20]. CHMp, derived from primary tumors and CHMm, derived from metastatic cancer, were maintained in RPM I1640 medium (Hyclone, SH30027) supplemented with 10% fetal bovine serum (FBS; Gibco, 1,600,044) and 50 ug/ml gentamicin (Sigma‒Aldrich, G1272) at 37 °C humidified incubator with 5% CO2. Two cell lines used in the study were authenticated, *Mycoplasma*-free.

### Exosome isolation

**Fig. 1:**
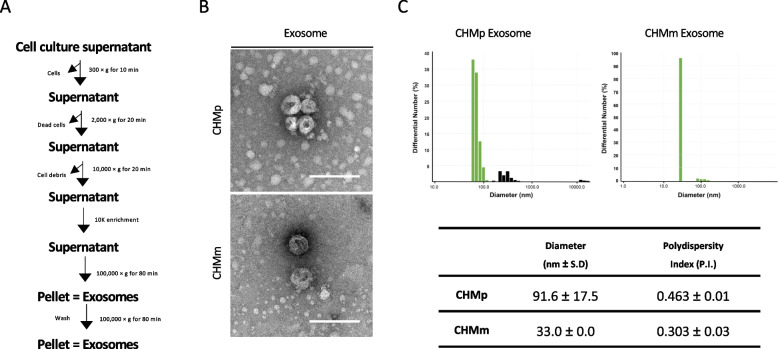
Isolation and characterization of exosomes. **A** Schematic flow of exosome isolation. **B** Transmission electron microscopy of exosomes from CHMp and CHMm cells. Exosomes are negative stained. Isolated exosomes show a cup-shaped morphology. Scale bar: 200 nm. **C** Dynamic light scattering analysis of CHMp and CHMm-derived exosomes. The mean diameter size of CHMp-derived exosomes were 91.6 ± 17.5 nm and CHMm-derived exosomes were 33.0 ± 0.0 nm

Exosome isolation was performed as previously described [21], and the isolation method is summarized in Fig. 1A. Briefly, CHMp and CHMm cells were cultured until reaching 80–90% confluency. Twenty-four hours before exosome isolation, cells were washed twice with PBS and then cultured with serum-free RPMI medium without any supplements. The culture supernatant (CS) was collected and subjected to differential centrifugation: 300 × g for 10 min to remove dead cells, 2,000 × g for 10 min to remove cell debris and 10,000 × g for 30 min. The supernatant was further centrifuged at 100,000 × g for 80 min. The pellet from ultracentrifuge was washed once with PBS and resuspended with appropriate buffers for the assay. All centrifugation steps were performed at 4 °C.

### Electron microscopy (EM)

Isolated exosomes were dissolved in PBS at a concentration of 1 μg/μL. Subsequently, 1 μg of exosomes was placed on glow discharged carbon-coated copper grids for 1 min. Excess liquid on the grid was removed using filter paper, and negative staining was performed using a 2% (v/v) uranyl acetate solution for 10 min. After draining the staining solution, the grids were air-dried, and they were immediately observed under transmission electron microscopy (TEM) at 120 kV. TEM imaging was conducted using a TEM Talos L120C (Czech) which located at the NICEM at the Seoul National University.

### Western blot assay

The Western blot assay was performed following a previously reported method [22]. Briefly, exosomes were lysed using RIPA buffer supplemented with 4% (v/v) 25 × protease (Roche, 04693116001) and 10% (v/v) phosphatase inhibitor cocktails (Roche, 049068545001). The lysed exosomal proteins were quantified using a BCA assay, and equal concentrations of CHMp, CHMm SW480, and SW620 exosomal proteins were loaded onto SDS-PAGE gels. The proteins separated by SDS-PAGE were transferred to an 0.2 µm nitrocellulose membrane (Amersham™ Protan™, 10,600,004) followed by overnight incubation with respective primary antibodies at 4 °C. Subsequently, after three washes with 0.05% (v/v) TBS/Tween 20, secondary antibodies were applied. After three washes with 0.05% (v/v) TBS/Tween 20, membrane was subjected to chemiluminescence detection using ECL (Biomax, BWP0200). (Primary antibodies; LDHA (expected molecular weight; 37 kDa), Cell Signaling Technology, 3582 T, 1:1,000, and GPI (expected molecular weight; 60 kDa), Cell signaling Technology 94,068, 1:1,000, Secondary antibodies; Goat anti-Rabbit IgG + H + I HRP conjugated, Bethyl, A90-116P, 1:3,000).

### Proteomics sampling and LC–MS/MS

Exosomal proteins from CHMp and CHMm (50 µg each) were digested with trypsin according to filter-aided sample preparation (FASP) digestion method [23]. FASP digestion was performed as previously reported in our laboratory [24]. Briefly, 50 µg of protein were mixed with 200ul of 8 M urea in 30 K Microcon devices (Millipore, YM-3). The reduction (10 mM of TCEP, Tris (2-carboxyethyl) phosphine) and alkylation (40 mM of IAA, iodoacetamide) of proteins were performed on 30 K Microcon with centrifugation washing. The resulting concentrates were digested with Pierce MS-grade trypsin for overnight at 37’C and desalted using StageTip C18 method. Briefly, The C18 stage tip was made by mounting three C18 discs (Empore, 2215) for reversed-phase material. Each StageTip was activated with sequentially 100% methanol, 80% (v/v) acetonitrile (ACN) in 0.1% formic acid and 0.1% formic acid. Next, peptides were loaded and washed with 0.1% formic acid. Finally, elution of peptides was performed using 60% (v/v) ACN in HLPC-grade water. Eluted peptides were quantified by BCA peptide assay (Thermo Fisher Scientific, 23,275) and 25 µg of peptides labeled with Tandem Mass tag (TMT) six-plex isobaric label reagent (Thermo Fisher Scientific, 90,061) following the manufacturer’s recommendation. The labeled peptides were fractionated into three parts using by SDB-RPS (poly(styrenedivinylbenzene) reverse phase sulfonate). The detailed protocol and buffer compositions were described at Mann et al. [25].

Liquid chromatography-tandem mass spectrometry (LC–MS/MS) analysis was performed with a Q Exactive (Thermo Fisher Scientific) coupled with an EASY-nLC1200 UHPLC system (Thermo Fisher Scientific) as previously reported in our lab [22]. The peptides were injected into an EASY-Spray column (75 μm i.d. × 50 cm; PepMap RSLC C18 particle, 2 μm particle size, 100 Å pore size) and subjected to a 90-min LC gradient at a flow rate 250 nl^−1^. The MS data were acquired in data-dependent mode and the full scan resolution was set to 120,000 at *m/z* 400. MS/MS raw data were processed with MaxQuant (ver1.6.5.1) software and Uniprot dog proteome (number of entries: 43,621) was used for database searching. Proteins identified with two or more unique peptides (> 9 amino acids) were considered significant. The false-discovery rates (FDRs) were less than 1% at global protein level. TMT intensity efficiency, multi-scatter plots and Principal Component Analysis (PCA) of identified exosomal proteins were analyzed using Perseus software (v1.6.15.0).

### Bioinformatic analysis

The UniProt database was employed to categorize protein based on their subcellular localization. Gene Ontology (GO) analysis was carried out using the DAVID functional annotation tool (v6.8) (https://david.ncifcrf.gov/tools.jsp). For network analysis, the STRING DB (v11) (https://string-db.org/) was utilized. Additionally, Gene set enrichment analysis (GSEA) plots of differentially enriched proteins were generated using GSEA software (v.4.1.0).

## Results

### Isolation and characterization of cancer cell-derived exosomes

To investigate differences between primary tumor and metastases, we aimed to use tumor cell lines originated from same individual patient. Unfortunately, no paired human breast cancer cell lines were available for this purpose. Thus we choose to utilize canine mammary gland tumor cell lines; CHMp representing the primary tumor and CHMm representing metastases, derived from metastatic lung pleural effusion originated from same individual [20]. Canine mammary gland tumor cell lines, CHMp and CHMm was driven by their natural occurrence and the gene regulatory sequence and reference genome of dogs is more similar to humans than mice [21, 26].

We previously established an exosome isolation method to purify exosomes from CHMp and CHMm [21]. The detailed strategy is outlined in Fig. 1A. To further characterize the morphology of CHMp and CHMm-derived exosomes, negative staining was performed, and transmission electron microscope (TEM) was used. The isolated exosomes exhibited cup-shaped membranous vesicles with sizes below 200 nm, and there was no significant difference in the morphology of exosomes between the CHMp and CHMm (Fig. 1B). Exosome diameter measurement by dynamic light scattering (DLS) showed that the average diameter of exosomes derived from CHMp was within 91.6 ± 17.5 nm, while exosomes derived from CHMm were approximately 33.0 ± 0.0 nm in size (Fig. 1C).

### Proteomic profiling and comparison between CHMp- and CHMm-derived exosomes

**Fig. 2:**
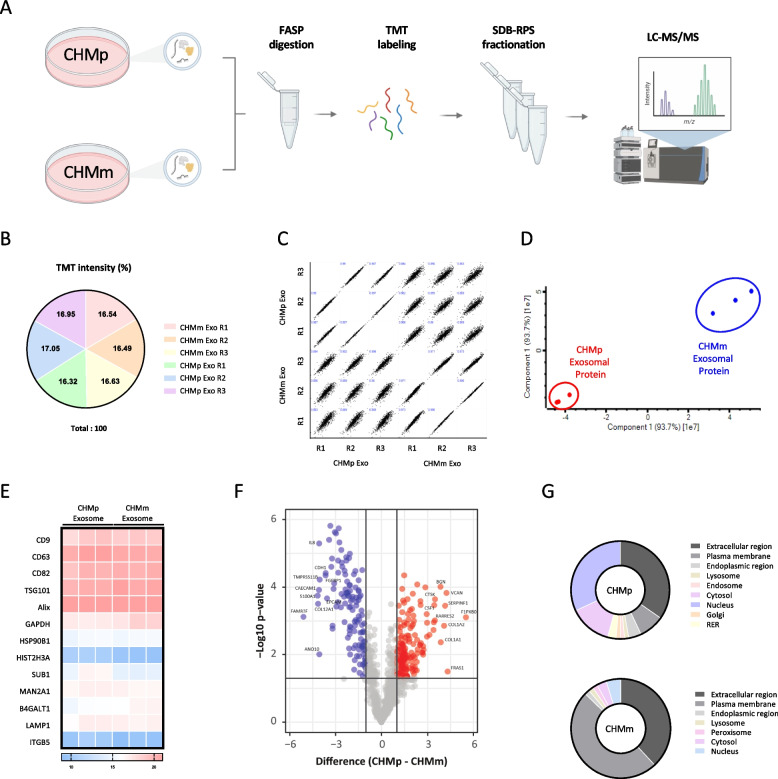
Proteomic profile of CHMp and CHMm-derived exosomes. **A** Schematic figure of exosome proteomics analysis. **B** TMT labeling intensity efficiency between CHMp and CHMm exosomal proteins in triplicates. **C-D** Principal Component Analysis (PCA) of CHMp and CHMm exosomal proteome replicates. **C** TMT intensity correlation of the replicates of CHMp and CHMm exosomal proteins. PCA showed high correlation for technical and biological replicates. **D** PCA showed high correlations of CHMp and CHMm exosomal protein triplicates. The X and Y axes show principal component 1 and principal component 2, respectively. **E** Heatmap of 13 exosomal proteins representing cellular localization. CD9, CD63, CD82, TSG101, Alix and GAPDH for exosome, HSP90B1, HIST2H3A and SUB1 for nucleus, MAN2A1, BTGALT1 for golgi, LAMP1 for lysosome, ITGB5 for mitochondria. **F** Volcano plot based on the Log2 (fold change) and their -Log10 (p-value) of CHMp and CHMm exosomal proteins. In the plot, red dots indicate the proteins that are statistically enriched in CHMp exosomes, blue dots represent proteins enriched in CHMm exosomes, and grey dots represent proteins that are not statistically significant. Significantly enriched proteins in CHMp exosomes compared with CHMm exosomes as control, Student’s *t*-test, p < 0.05, obtained in Perseus software. **G** The subcellular localization of CHMp and CHMm exosomal proteins showing significantly differential expressions. Predicted subcellular localization were obtained from UniProt (https://www.uniprot.org/). CHMp exosomal proteins were predominantly located in the nucleus and extracellular region. *n* = 3 biologically independent exosomal protein isolations. Figure 2A created with BioRender.com

Next, we analyzed the proteins within the cell-derived exosomes. The proteins isolated from the exosomes were digested into peptides using the FASP method. Technical replicates consisting of three samples each from CHMp and CHMm, were labeled with TMT, fractionated, and subjected to LC–MS/MS (Fig. 2A). The exosomal proteins derived from CHMp and CHMm were analyzed in biological triplicates, and the TMT intensity for each replicate was uniformly labeled across all replicates (Fig. 2B). To assess the correlation among replicates for the CHMp and CHMm exosomal proteins, we performed principal component (PC) analysis which demonstrated a high correlation (> 0.9) between replicates (Fig. 2C,D). The exosome markers CD9, CD63, CD82, TSG101, Alix and GAPDH were enriched in all exosomes, while other markers such as Nucleus (HSP90B1, HIST2H3A, SUB1), Golgi (MAN2A1, BTGALT1), Lysosome (LAMP1), and Mitochondria (ITGB5) were not (Fig. 2E). Out of total 1,284 identified proteins, 87 proteins were enriched in CHMp exosomes by log2(fold change) > 1.2 and *p*-value < 0.05 and 65 proteins in CHMm exosomes by log2(fold change) < -1.2 and *p*-value < 0.05 (Fig. 2F, and Table 1, 2, 3 and 4). The 87 CHMp exosomal proteins were predominantly localized in the extracellular region or nucleus, while 65 CHMm exosomal proteins showed different localization patterns, mainly in the extracellular region or plasma membrane (Fig. 2G). These results suggest significant differences in the composition of exosomal proteins derived from primary tumors and metastases, indicating that heterogeneity between primary tumors and metastases is reflected in the composition of exosomal proteins.

**Table 1:** Total protein list

CHMm exosome			CHMp exosome			
Reporter intensity 0	Reporter intensity 1	Reporter intensity 2	Reporter intensity 3	Reporter intensity 4	Reporter intensity 5	T: Protein IDs
68688000	82287000	90220000	7181300	3743500	3419500	J9P6G0
47075000	43429000	44312000	42513000	44483000	26874000	F1P6H7
20077000	13621000	13659000	2437400	2639700	2556700	F1PR26
16691000	20835000	21783000	6099700	5178500	3175600	F1PBI6
14508000	17116000	16416000	61093000	36186000	32907000	CON__ENSEMBL:ENSBTAP00000024146
12816000	10221000	10520000	4899700	6556300	6307700	E2QYU2
9915000	2813100	3985000	4782200	6150400	3327600	CON__P04264
9836700	14998000	14928000	42994000	25855000	22428000	CON__P02769
9283100	7287700	8472700	3276100	6333100	5300300	E2RT60
8956000	6587800	7569400	5772300	9575700	9469400	F1PFG6
8707700	7214500	8044900	5167700	9514600	9091400	E2R0T6
8678200	7158000	8043300	2396600	2891600	2323900	F1PCD8
8124600	6091700	6773500	1032200	1026500	883550	F1Q2Z6
7537400	5314000	5337400	5880500	7419200	6886100	F1PFZ5
6908700	4620400	5113600	1831000	2437400	2297400	F1P8D5
6590800	5475500	6246700	1812500	2080900	1751600	F1PHK9
6147100	5064800	5384900	2737800	4100000	3473200	F1PEZ4
5920600	4391300	4929000	399890	272680	265770	F1Q3B8
5777600	5379600	5875000	1660300	3380000	3433100	F1P6B7
5725600	7779200	8818600	9514200	10872000	7207500	J9NRJ0
5312900	3783300	4114300	5488500	11304000	10656000	E2RQ81
5285800	4554600	4942500	1138000	1245100	1145700	E2RPP1
4959800	4323600	4854300	1029300	1386300	1359800	E2R9S7
4956000	1430600	1930900	2157700	2686600	1241800	CON__P13645
4767600	3886200	4102700	1187200	1497400	1436300	E2REA9
4729600	3418600	3855500	442800	388310	397880	E2QW13
4661700	4088900	4590000	1586500	2256600	2085900	J9NZA9
4657000	3699600	4346500	1325800	2049700	2002900	F1PBL1
4617800	3849000	4437400	787680	1058800	1011100	E2R830
4262000	3521000	3147900	2062800	2965700	3103100	F1PQM7
4253400	4948100	4644700	6971400	10378000	10693000	E2RKQ6
3914900	3203300	3510700	679510	1526900	975570	F1PWE1
3909900	3313000	3657300	1124000	2140100	2008300	F1PHQ0
3760200	2841300	3068200	1412100	2459800	2252500	F1Q439
3699800	2909000	3303500	3150500	5843800	5769700	Z4YHI2
3669400	2238000	2522000	1672000	2537600	2199200	F1PKR0
3563900	2659800	2877600	3617500	7528600	7187100	J9PAI4
3559900	6243400	6532500	15322000	7164100	8389300	CON__P34955
3554300	5081000	5141800	18264000	9126400	7332100	CON__Q0IIK2
3544500	3692900	4129800	4729100	6571800	5819600	F6XXM1
3495400	1190100	1602600	1648100	2318500	1080500	CON__P35908
3463500	2910300	3295800	1867900	3964300	3728200	F2Z4P4
3413800	3339000	3637100	730450	872120	794250	E2R0R3
3357300	3308300	3615600	3230900	5894200	6013900	E2RLS3
3228800	2125400	2297200	2484300	6933400	5552100	E2QWY6
3205300	2510100	3041600	350930	214750	198630	F1Q1M9
3185100	3499500	3872600	5156300	4002400	3542100	CON__ENSEMBL:ENSBTAP00000032840
3138300	4057900	4319200	16300000	8668400	7732700	CON__Q2UVX4
3132800	2724200	3211100	988450	1838000	1759200	F1P719
3036000	2110200	2353200	474950	532160	510850	A0A140T8E6
2961800	2224500	2398500	3166200	6633900	6137200	F6V0D8
2920400	2358400	2628400	493200	739170	628210	J9P0D9
2905700	2980300	2966000	2612700	4311300	4021700	F6XRY2
2896400	4222800	4577900	6186300	5329400	4565200	E2RLQ9
2848900	2773900	2738800	3891900	7121700	7141400	F1Q2F6
2822300	2162800	2489800	1174700	1669700	1482700	F1P8Q0
2813100	2329000	2558400	922540	1187200	1095700	Q2KM16
2783100	2359800	2816500	1591200	3495200	3172300	F1P914
2739600	1889100	2085300	2851800	5666500	5010300	E2RLL6
2590000	3134600	3169600	6920400	5435600	5562500	CON__P15497
2450600	2229700	2489900	241240	217850	247690	F1Q3A2
2449300	1762600	1857800	2447000	4528800	4075000	E2R0L9
2421100	2653200	3295100	263720	198760	206080	F1PAA4
2414100	3083400	3367600	8136700	7852900	5739700	CON__P12763
2400800	2176300	2705000	10223000	7778000	5795700	G4V2B6
2395400	2177700	2331600	1136700	2474100	2150000	F1PTZ7
2224000	1988200	2235500	1038200	2002100	1958300	J9NV93
2110900	2104700	1990300	830470	813110	593720	F1PPY4
2063500	1449500	1688500	989630	1334800	1266800	E2RGP6
2051500	2047700	2428600	743230	987880	986510	X5IHJ5
2019500	673020	981690	1068200	1362900	871580	CON__P35527
2008600	1457200	1647400	894310	2357400	2180500	J9P6X5
2002200	1396500	1627700	949580	2327600	2080500	F1Q0Z2
1969900	2110600	2277900	333500	319920	292810	F1Q2N9
1934700	2113400	2223000	212820	140680	152410	E2RLM9
1891300	2687700	3274500	1220100	1939700	1213800	J9NZ79
1852600	1097600	1189400	783340	1614700	1440300	F1P7B0
1849400	646020	866580	903400	1075300	631560	E2REU6
1830600	1749300	1866200	1011900	1533300	1677800	J9P730
1793900	1247000	1434600	1952700	3768100	3183400	F1Q331
1767100	1300200	1393000	1636700	3166900	2857300	F1Q406
1735500	1333400	1499500	1053800	1832400	1652700	F1Q0H3
1732800	1674600	1815700	1148400	2809200	2720300	L7N0B2
1717600	1349000	1559000	641100	1040500	939250	F1Q4C5
1710900	1228900	1389500	1072300	3600200	3132300	F2Z4Q5
1710400	1349000	1614900	2201500	7621400	6461600	J9P6S8
1691400	1351300	1364900	1383700	5360200	4717100	F1PX67
1687300	912710	633070	114950	118500	92605	E2R5S3
1646900	1234700	1215400	468120	553950	462520	F1PP89
1592800	1265500	1342600	1579100	3075000	2951200	F1Q3V2
1592400	1804100	2005400	1477400	3254700	2442400	F1P9J3
1559500	1703500	1786600	1112200	2357600	2188000	F1PHS5
1542500	1352100	1481100	1074600	1101800	1055800	F1PEN6
1540700	1495100	1385600	1352200	2784100	2887900	F1PIT4
1535900	1389800	1578300	875420	1288700	1201900	E2RSI6
1524900	1237000	1356200	805320	1388200	1270600	E2RD95
1517900	1715900	1910700	362700	414900	463850	F1Q4F9
1513000	1305200	1597300	122710	62984	70028	E2RP88
1493200	1036200	1260700	862960	2326900	2032200	F1Q424
1472700	1119700	1178700	283030	313010	285290	J9P7H5
1470900	1728100	2007900	6302100	6582100	4719900	F1PHX8
1458900	1079300	1083800	2025800	3313500	3495500	CON__P01966
1455200	1036500	1168500	827240	2616000	2478600	J9P425
1434500	1951200	2061700	4141900	4378100	4540300	CON__Q3SX09
1422400	1155600	1278800	2043000	3859500	3396000	F1PCH3
1419500	1059700	1147800	1394400	2979700	2847800	E2RB79
1390200	624730	664570	538090	687340	559190	F1P7Q4
1389800	1054000	1212000	797840	895910	674560	F1PKH0
1354200	2046100	2129100	6966700	3038100	3160800	CON__Q3SZ57
1336800	1676300	1969400	1323000	1585200	1375300	E2RJJ6
1333300	1048500	914700	1319000	3115800	3100400	F1PTZ9
1318000	806530	903760	898320	2409700	1756100	E2R8R8
1318000	1078200	1231800	330720	391220	439210	F1Q1C9
1299400	1264600	1382300	2157500	2770100	2672000	F1PM26
1295600	1009800	1100300	1393400	795820	750610	CON__P41361
1270700	1954100	1910800	4860500	2354900	2532600	CON__Q58D62
1243500	983570	913020	1825200	3540600	3499800	F1PBT3
1241800	1088200	1273400	569000	1111100	1016400	J9P9V8
1229500	884680	982770	462270	875400	839490	E2RSF6
1229300	798270	856310	561060	869280	525430	M1VEH7
1203200	1834900	1910100	1838600	3275200	2225100	F1PPA1
1196300	916660	996200	713850	2059900	1930700	F2Z4Q1
1195600	1228700	1264400	1379100	2275500	2396500	F1PGY1
1171500	1193700	1245900	386600	833920	798350	J9NWJ5
1164200	863950	1033400	484430	1354600	1151700	E2R546
1159800	1388800	1350900	579230	1122500	1210700	F6XY66
1146500	771230	812760	164640	255180	234950	F1PKV5
1143700	1081400	1199200	157670	143570	135870	Q4W6L5
1143000	1170200	1094000	973650	975450	996820	CON__P00761
1139800	898370	1040800	3267200	5040100	4690800	F1PVW0
1132600	2247700	2476200	4394200	4612300	4408700	F1PUI4
1126900	946180	1108400	1226400	4172600	4076800	L7N0L3
1124300	840460	978120	629830	2226600	1950700	F1PUX4
1120100	977170	1105200	1972500	3963700	3553400	F1PHR2
1113200	1101200	724910	2204400	1390400	1773500	CON__P81644
1108800	922460	1049700	437590	655630	598110	O97702
1090500	1042400	1239600	687990	1592000	1457400	E2RKW9
1080900	1013100	1269100	796720	664220	474550	F1PMN4
1063900	680480	674340	301860	515610	395140	J9NRH5
1054600	1362700	1428700	6060300	3457300	3368000	CON__Q9TRI1
1052300	899260	944510	1171100	2433300	2355400	J9JHE4
1041700	1224400	988190	511510	1219800	997590	J9P4E7
1040600	1328900	1468300	295100	367750	366910	E2R2V6
1039100	985280	1115400	536400	842740	814620	F1PDQ4
1026400	1284700	1317400	4196200	2321200	2064800	CON__ENSEMBL:ENSBTAP00000037665
1018900	913250	1053100	923890	1763500	1766300	E2RHG2
1012400	1074000	1178500	1054300	1618900	1752800	E2RC20
1009600	908730	1070900	986440	2050300	1685200	E2QZT4
1006100	824910	820520	1155200	2922400	2591100	E2RSR5
1001800	839830	1010900	377380	686250	630480	E2RBL9
998470	926260	1117200	492000	639680	573710	J9JHQ1
982610	861600	972450	630260	1206500	1177500	E2QSF4
977510	1487500	1649900	3845400	1929700	2025700	CON__Q3SZR3
975780	1117900	1247400	3376400	4783700	3949000	F1PU95
962850	1127100	1310300	642520	1170600	1082900	F1PDR0
940490	1406600	1648000	5253600	7804400	4769600	E2RCV4
935440	969920	1041300	1609100	3420400	3194200	F1PGY9
932360	962720	1039100	2646200	2949500	2558300	E2RTL4
927560	683800	793090	689240	870580	746130	F6X907
926910	756020	896080	325170	505830	479530	F1PQ93
918740	588010	590810	380870	1183100	1044400	J9P1D0
908540	815060	935300	697020	1163800	1070200	E2R2K4
875960	479960	429840	440770	1717700	1256500	J9NRJ1
872060	964460	1095100	1041800	2106100	2204100	E2RRC9
872000	1387000	1450100	3640800	2444900	2385800	CON__ENSEMBL:ENSBTAP00000018229
847850	940070	1139000	225390	229350	241110	E2RLA5
844450	835130	1044600	840890	1034100	987040	Q56JK3
837170	871820	997690	1244500	2106400	1993900	F1PSC2
830750	907560	1050000	2689400	1568900	1428700	CON__P01030
829940	608660	568040	396190	1225800	1080200	E2QYD8
827270	577560	669710	366810	960070	822580	F1PI16
824750	672750	812570	991310	2057300	2022300	E2RCI8
822880	694920	661270	240420	359770	364550	J9NZ45
818900	867860	888880	1403200	2417000	2526500	F1PLT8
815770	883990	968460	890900	1461600	1702500	F1PMP0
814710	619160	705480	585260	1491700	1247600	F2Z4P2
809920	805720	753750	703450	1687800	1609600	F1PCI2
794140	696460	820980	499980	833040	756350	E2QZH1
790090	801900	879000	2186000	3870300	3214400	F1PWW0
787670	1128100	1238400	4130200	2013000	2030200	CON__Q3T052
783750	777450	930510	456360	800500	775580	E2R5G6
782340	573150	621320	551360	1714900	1427900	E2RFR0
780470	634480	693210	69531	63925	62778	E2RP76
769570	792050	903090	454300	634580	627500	F2Z4P9
765330	858740	850310	724770	1353000	1365200	F1PPT7
761900	508500	594560	283420	378480	343320	E2QZC7
755860	523210	597640	490660	903150	843870	E2QXN8
754100	704250	788920	732770	555780	419670	J9P8M2
751330	834030	826570	1221100	1288000	1221800	F1P8T3
741340	525030	592470	223670	329490	303430	F1Q1H3
736960	683870	805750	566140	729950	678220	E2RMT2
710010	558880	644560	899970	2367500	2367900	E2R6K5
701250	745710	758090	1184500	2166800	2119500	F1PUB5
697840	617840	687050	392450	556430	542880	F1P8L7
693670	747020	788410	493640	537570	555060	F1PML2
681900	820330	859330	1964700	1449700	1213800	CON__Q1RMK2
676040	519240	540020	496660	1420200	1231700	F1PLR0
666900	472080	467360	560380	885290	822120	E2RMT4
666810	606150	708420	412380	551220	521060	F1P6Q0
666400	822690	567810	661480	1198100	1391600	J9P4F3
665780	473300	523020	328810	951670	796550	J9NU88
665340	653460	753630	513520	846710	830340	E2REK6
664230	460530	498190	305580	841510	861650	Q9XSU5
663060	455050	509000	394040	1192700	1098000	J9JHJ2
661940	823220	975640	2160800	2680100	3605700	E2RMA3
645280	1139300	1015600	1200200	846790	858910	F1P8Z5
642490	609070	683500	732850	1186600	1042600	E2R4X9
637170	600450	713610	301430	456370	452450	J9P6N4
632840	561610	628370	548450	1026700	749940	F1PWC7
626620	447030	519950	135690	144870	110920	F1PW10
625890	685720	724640	244070	423670	458180	F1PWN2
620610	662970	718950	1402800	2248800	2316100	E2R761
619350	413850	448800	528230	911840	765690	F1P679
610640	485950	552200	202040	298910	296380	E2RPE3
606750	497390	551880	366410	598720	591100	J9NTN5
601900	464750	500000	670000	1913600	1631300	J9P8R0
600550	738330	865890	165020	461090	255670	E2RAA0
592120	515880	579870	283970	373210	360140	E2RBB8
586750	700510	742770	263620	334270	373860	E2RJH2
582600	565730	634360	81132	86024	89927	F1PHN5
581400	673010	698800	1570000	1551400	1408700	CON__P00735
571770	442800	506920	570200	977970	786370	F1PB66
568600	862570	968570	134840	123240	114990	F1PRB0
567790	510230	582360	407700	665550	665870	F1PKI4
564180	642080	692080	1117100	2092600	2190500	E2QY07
561640	1046900	1182300	2801100	1440700	1600000	CON__Q9TTE1
561040	499340	554250	256310	425220	436090	J9P9V0
553320	357000	353690	144550	472150	425430	E2R0A6
550840	476650	458300	813230	1408300	1396000	J9P9G4
548000	401810	498330	658560	548150	359930	F6XL96
544890	648830	728940	345150	590350	663820	F1PC59
544360	459460	495820	615680	1419700	1310500	E2R1J6
540980	329260	393610	225490	677410	559180	E2R4F5
540790	452770	528380	606660	2110500	1933700	J9P6J3
540100	677620	731530	225640	366670	344560	F1PDP1
539800	528710	577320	451010	644450	601580	F1PSC1
537970	844640	793560	82032	76788	84838	E2QXH3
532810	440560	525630	220160	304080	299210	F1PPL6
528500	415680	484660	229700	364900	328120	F1PHQ7
525010	556380	638650	730260	1632700	1572600	J9P969
523830	428430	522550	102600	138320	130370	J9P7J0
512880	595490	595190	471650	748950	743690	E2RIV1
512410	423300	486070	256670	1099800	916880	E2R9V0
502890	695160	815220	279860	385450	398390	K7ZSN9
501380	491710	552950	969680	4137500	3926700	F1Q0B0
496720	396630	415610	264870	936450	908380	E2RS49
490560	418990	479260	325600	365120	405910	J9NWL5
481200	640900	626440	1135100	1072800	955370	CON__Q3ZBS7
479910	644920	1022300	10764000	6919500	4808300	F1PG08
476380	427050	455910	623440	1370700	1283400	E2RQP6
475310	453620	508010	293880	457700	441990	F1PV63
472470	522890	588440	1711900	3807700	4597900	F1PLS4
467330	410930	510090	126170	98841	93689	F1PKT2
465520	444810	532820	650310	907690	832430	F1PY05
465200	375550	382520	166550	483280	424920	J9NUD9
462990	514320	541560	472070	835270	927520	F1PAG6
462030	679090	770750	2489400	1527900	1398100	CON__Q0V8M9
461780	434470	481850	523430	1061400	1018200	E2R5N5
459640	241830	249930	246450	601610	466830	J9NZX6
453600	594590	606970	379630	776970	726660	Q6IM72
446050	381220	399900	647610	1181600	1109900	E2RBR6
444160	392380	410130	421740	756210	751320	E2R985
440640	372640	407390	114850	125990	108910	F6V9D7
440310	470940	531970	229620	277760	346060	F1Q4G4
437760	477410	537090	964090	666860	500790	CON__P06868
436430	436620	523950	199430	306300	324320	F1PKW7
434540	555290	482670	1391000	793420	671940	CON__Q1RMN8
430390	397860	442960	471580	919330	859660	E2RCP9
427800	663920	686540	1849500	1335100	1307800	CON__P17697
427100	303400	312940	207950	439360	384030	F1PGD7
425570	460830	514860	310550	501700	507110	E2RCY1
420580	508760	440950	237300	473110	625450	F1PQN5
419340	447900	469970	640130	2185400	2010200	E2RTB1
418870	467230	508710	290110	401680	422350	A0A222YTD8
418000	280920	280700	215640	616600	513450	J9NVM6
417890	312840	374510	257700	389190	367690	J9P003
413050	333200	338610	638470	1389400	938470	E2RMN2
410850	330150	367460	562650	1318600	1165300	F1PKF6
410200	387870	434430	72519	67272	60533	F1PF63
407090	363290	440130	79886	87697	78972	Q9TU80
406410	300430	342590	187220	266130	250910	E2RP16
402080	255710	277300	194710	640850	403290	F1PF85
401700	235170	261660	109440	335740	267460	E2R118
397180	574840	595230	356960	422660	456350	J9P3J4
396340	406970	377230	447720	1026000	1039100	F1PKS1
395580	303770	356790	513380	1733600	1483400	J9P604
393800	404910	464760	334310	652750	609720	F6Y6T1
393790	287480	321340	361210	959870	853140	J9NRJ3
393010	628230	701820	137190	199060	185060	F1PW98
390560	233240	138840	255600	566560	519860	F1PDX9
388570	375280	400840	362940	422810	382600	CON__Q28065
387930	339770	346320	622380	1043300	971180	J9P849
385470	364740	371180	188110	288470	267870	E2QUU5
384840	413510	506490	96629	75516	82362	F1PFM5
380420	341620	364250	175290	251910	198140	F6V9R9
378010	348640	357770	557420	1040100	1023100	E2R4H4
377130	255580	259730	253740	692370	575530	F2Z4N5
375960	382820	406850	742430	1365200	1019600	E5Q8W5
374590	458100	497560	105080	200600	194880	J9JHV7
368280	392890	335750	384600	1548500	1381300	E2RNB0
367810	359730	301890	135270	253930	271460	F1PFF2
366370	365520	436170	265320	439500	421120	F1P9S5
365600	338170	372310	46675	40102	43628	F1PTX8
363650	450910	599390	67658	70874	54733	J9NTX8
361220	317330	331500	194150	394880	402420	J9JHN1
361180	547810	548550	2163000	1284000	1234800	CON__Q3Y5Z3
360360	292130	347970	385720	554640	490860	F1PB20
359690	256000	280150	157680	213160	192200	J9NTN8
355350	317940	279550	306660	908210	872360	E2QZ50
353980	333780	336570	528780	936310	875640	E2R0B6
353540	304930	334680	423530	747470	681600	J9P326
350630	367210	422970	1210500	1803000	1485700	F1PLA1
348210	549380	711350	286890	300570	268600	F1PXX5
347540	290790	315130	230890	483640	447580	H9GWA4
347110	611150	643930	123320	119550	121300	E2RNC2
346970	368930	422350	238800	309680	286960	E2RKC6
343200	325940	381110	105040	144860	161180	F6Y4A3
341490	372040	418920	1008300	635730	572330	CON__Q1A7A4
340660	486040	519760	77626	85648	92658	F1Q0N9
338910	384430	395170	417090	698110	714590	F6X637
335160	332480	358700	535010	667700	640060	CON__Q28107
334740	197170	192200	125250	221560	181330	F1PNT6
328210	378690	382650	292770	980610	1069000	F1PA19
328100	232380	248510	172100	378890	320360	J9PAU6
327530	261780	278440	212220	891370	796450	F1PAJ4
325730	242540	249240	188040	656240	538460	A0A0A0MPE0
325400	327980	356050	56279	73739	67707	F1P8R9
325090	358900	428070	68095	68859	70360	Q867A2
323310	306670	354970	337030	621300	582690	J9NY67
318850	312030	349020	397220	801430	781580	E2RJ26
318630	210210	208080	179140	727440	651360	E2RIQ8
318390	281450	335080	132940	223120	202970	J9NX28
317220	382760	397920	393770	685040	688100	F1PCQ3
315510	270680	308550	144320	283810	293500	E2R507
314880	412070	474850	697110	1402700	1254300	E2R6Q7
314370	338730	345150	559790	1126900	1130200	E2R3R2
313520	321100	374320	284700	935790	862000	E2R8A8
312120	354540	533730	5808000	9180400	7361600	F1PGL1
310480	290110	315860	256720	502730	492500	E2R002
307710	253700	288530	347900	684130	631180	E2RR68
306610	343550	435820	532210	2397900	1658400	E2QYP1
305950	252680	286520	156340	254450	225190	E2RHL4
304660	254360	272230	122050	200720	197730	F6X9C1
303890	293810	243840	39498	27001	32941	E2RJT2
303700	434720	448130	1278600	575780	652120	CON__A2I7N3
303680	321390	342340	320390	541470	545490	E2R574
302950	226350	245520	156700	249640	234570	F1PH57
302290	265430	300370	504100	1025800	1040100	A0A346M2B2
301720	468360	558930	669360	904030	792280	J9NZK5
301190	262310	292250	223050	408840	342040	F2Z4N7
301080	431760	497010	1301900	778020	797220	CON__Q3MHN5
299400	399440	464260	1602700	622120	599570	F2Z4Q6
298140	221650	259400	193340	679410	558190	J9P9Z7
296550	424470	446430	1325200	873220	711340	CON__ENSEMBL:ENSBTAP00000024466
294140	262490	300070	198140	257580	240410	F1PHV7
292820	285530	320280	150780	262770	270820	J9P5N6
290120	253820	277520	416760	558010	531730	E2RAV2
289740	298320	344210	467440	822410	874960	E2RT65
289070	316410	375770	431200	1392000	1190700	F1PGB6
285250	291720	389260	2221700	5479000	3159400	F1PLV2
284790	254400	287230	388290	716110	653350	E2RQC9
284580	123170	99700	70503	169930	134760	J9P6P5
283270	269770	332180	205320	238930	239800	G1K268
283180	421780	295840	264500	298220	262760	CON__Q28194
282530	425910	493370	637910	506500	455780	J9P5W4
280260	276560	281280	320110	549980	562960	F1PE28
279150	296820	317020	340020	766740	827160	F1PBZ4
278280	385010	701070	12421000	5243900	3536000	F1Q3I5
277220	215520	250530	89371	94671	88474	B2KN54
274980	242370	290100	122990	211350	201410	J9PAF7
272590	259600	324650	533520	2234000	1791400	J9P3D2
272040	227410	223600	134800	252350	251530	G1K288
271480	265500	321370	152920	226390	220520	F1PSK6
270210	251180	275150	157910	263960	259280	F1Q3Y0
267200	249880	277970	169450	323830	328800	F1Q264
265720	221180	250120	80183	93689	89551	F1PWL4
265350	219850	248140	311350	329050	336980	F1PV82
262940	299010	315490	366300	1303500	1081900	E2RIX2
260050	256660	283840	105790	114880	114680	E2R8K5
258790	465720	545600	172790	410350	453110	F1PEQ0
257460	326970	233420	1440900	2635800	2334800	F1P933
257280	217270	247090	298520	864960	696480	F1PSI7
257100	369190	413360	882780	745910	723950	CON__P02672
256740	213260	254070	57639	45628	49945	F1PQT5
255870	364000	440700	950950	2572400	2018400	E2RGR7
255090	199180	249390	143530	550070	459800	E2RSU0
253640	210020	247430	177850	368190	357420	J9P966
252040	155680	186220	69940	100530	88850	E2QUQ7
248290	205950	234230	159820	214240	193240	J9P0F9
246300	205480	249040	449800	769560	608540	E2R806
245770	128690	142030	47062	52327	28893	CON__P02777
244310	177640	195860	86434	277280	220670	E2RI34
243240	222370	240240	155260	455920	257800	E2QXF0
242400	219200	244430	191350	209970	192230	J9NWI0
242070	198850	211590	190540	460120	319350	F1PZ83
240260	220070	238240	357890	743190	723990	F1PU93
239820	265290	297820	266170	516400	521060	F1PQS3
239760	223080	252180	29134	37230	34095	F1P9B6
239540	270040	367720	354760	712190	509720	E2RFV7
239220	254220	274340	319490	395840	392370	F1PL97
239160	244310	256750	307080	734510	706320	J9NTR8
238510	204360	230680	98403	136470	136940	E2QUB4
237870	276110	322350	268720	450560	454330	E2R7F1
236070	201090	226530	183590	423830	408450	J9NUP0
235740	230680	245830	300830	557760	536040	E2QWG8
234770	201150	230460	174090	273870	240400	F1Q0S2
234170	258340	305760	170020	310460	303510	J9P735
232220	226600	245080	313500	559470	550270	E7BUQ0
230320	287250	317140	579010	720320	600420	E2R8D6
227700	220530	261620	288970	486550	455800	F1PZD5
226560	191760	205620	466180	610850	513400	E2QXS7
225100	237650	254340	414880	445060	425960	CON__Q03247
224970	249290	244320	74104	139080	128410	F1PPA0
222220	218900	227130	348400	638390	663090	E2QW34
220480	406630	417080	147400	187390	187280	REV__E2RCP3
219270	204840	242630	147200	249860	241360	E2RRC6
218780	151290	157940	257220	785090	779660	E2R1A0
218000	214340	227130	384150	616400	577720	E2QX17
217880	197120	242200	156210	206900	199440	J9NUL0
217480	162960	179080	199180	357110	343410	E2QWU0
214990	170760	207790	170950	289660	283840	E2RM09
213350	196050	209270	100980	140250	138860	F1PDS3
212320	219610	261120	149810	226400	229790	F1PLT7
211800	203480	199990	570300	223670	220900	J9PBN6
211290	219920	245680	131670	322720	281880	L7N0I1
211090	195370	210640	145570	286670	423690	F1Q433
209970	220160	193130	158800	174310	123860	E2RSX2
209580	178980	205100	88472	129740	120680	F1PVZ9
208860	185750	234030	101980	124650	113760	F1Q136
208010	240120	247430	251160	584050	514620	E2R925
207420	258200	304560	176980	385140	398780	B8ZXI2
206990	181000	196420	218300	396250	397490	E2QXY7
206740	210750	222170	271760	531770	526010	E2R9A2
206730	198390	202520	258390	375820	388600	J9P6R1
204160	186480	206760	240230	560090	498170	J9NRI0
203550	311900	349740	869440	441080	430260	CON__Q3SZV7
202010	201550	241060	796670	473000	383460	CON__P28800
201310	207570	244500	234090	510270	474710	F1P969
201230	175120	212460	23617	18816	20311	F1PP71
200290	237030	270010	199240	355250	365150	E2QZD4
198980	198140	217080	225290	184920	156030	F1PWR1
198980	125780	127380	116690	648450	370630	E2R4N7
198200	187010	214970	129930	479120	425210	E2RB34
197580	200690	239730	152080	237720	190680	CON__ENSEMBL:ENSBTAP00000034412
196400	189070	217580	87337	135640	133890	F1PTG9
195350	241040	263880	300000	1066200	948170	Q32KH2
194120	244140	285550	137650	184430	145620	E2QYZ4
193970	209160	236970	117580	187640	189500	J9P743
192290	145540	171940	60250	92540	95839	E2RLV2
190840	236690	240340	681820	461860	466830	CON__P01045-1
190740	281450	323620	365160	338530	276830	J9NSQ6
190560	157280	190720	220540	183660	199470	E2RDX2
189470	154760	164420	187300	327040	315730	E2RD65
189090	161160	166080	230900	443490	403460	J9P4R4
188320	180190	201100	133750	213010	227440	F1PE09
188160	127080	152610	180670	225950	201320	F1PAF1
188020	166210	168510	179380	388160	349240	F1PQ40
186880	159270	166470	135140	127440	120650	F1PWA9
186800	184380	205670	161340	363920	344900	F1PR82
186290	118040	141280	56868	190100	157440	E2R149
185690	211760	232870	111250	180340	180470	E2QX57
185670	119440	144870	122250	445930	374500	F1Q1T5
185270	286480	319600	251970	72812	125550	Q9MYV0
185220	169770	185270	183650	246120	250120	E2R0I9
180420	234170	91392	333100	813940	960150	F6UXU9
179740	147610	163500	295240	549790	505890	E2RHX6
179490	84127	94878	51606	159810	96549	J9NUU3
178600	142920	144250	204440	435760	379130	F1PHR6
177360	228650	408760	6873500	4116400	2811400	F1PHY1
176130	204190	205120	76302	96011	97972	F1PDQ0
175070	128600	147460	142800	386140	284460	J9NXE2
174690	164920	197780	153340	209430	213150	F1PR93
172570	155590	184110	129320	390890	364400	E2R9Y9
172030	122720	136290	67035	165360	149560	E2QUE7
171970	166130	183110	239820	389990	375900	E2R9J8
170200	162740	184640	405960	767640	704370	J9NWI6
169490	206670	231270	194840	347070	397690	J9P7Y8
168330	164960	176230	112790	183780	193400	F6XNH4
167800	180990	184410	109420	142310	143930	E2QRR1
167250	154100	190990	69136	271590	232230	J9NYB5
166920	157900	183760	138540	318410	289360	E2QYC2
166790	143960	157980	110000	214050	216350	E2RJU3
166350	200410	239690	524580	712870	659060	E2R1I9
166180	206360	226490	501360	428540	371000	CON__P02676
164330	148880	173720	117990	180290	168060	E2QU34
163970	142220	158390	92954	156900	153320	J9P652
163480	132150	155460	149480	410710	350310	E2RCZ6
162040	207100	242930	50159	69357	63185	J9P6J1
160770	237630	278570	663940	385130	354120	CON__Q2KJF1
160630	242730	268390	572660	828300	900530	F1P9E5
160440	145590	173130	79588	111160	103930	F1PNV3
158730	147020	223090	126570	204460	216520	E2RN10
155610	178020	189470	221190	404090	418130	E2RF46
155260	146070	166610	185650	550400	461130	F1P7J0
151890	133180	149270	124820	220350	212980	L7N0D0
151230	172610	202080	797950	852490	953510	J9NS23
151060	144540	162680	85260	111980	101870	F1PUX3
150600	159280	145570	360180	321400	330330	CON__Q3SX14
150260	131460	146180	43702	55783	52353	G3FJD3
149520	161410	179390	112070	185560	185700	E2R4I1
148760	143270	167850	120210	241040	262690	F1P6P2
146850	118720	125070	163330	485250	398030	E2R1C4
146780	92139	106030	67649	146270	112630	F1PWM2
145790	128020	126510	72314	138560	118760	J9NYK5
145400	156790	167390	154430	278910	286140	L7N071
145160	184970	204950	202800	942460	911420	E2RMN6
145070	127720	139440	170340	373300	339080	F1PU02
144200	124940	145180	151140	365060	311650	F1PUB9
143680	127030	142520	76590	106620	103550	E2RC10
143560	232160	265090	39490	34204	43274	E2RT70
142350	142330	162330	201310	840860	744500	F6XIK8
141530	136500	163940	73643	79536	83582	F1PWT3
140900	170560	139750	549300	455650	446620	F6UME0
140450	144310	145400	128740	367790	360540	J9P5T5
140200	132010	163890	217450	270280	261790	E2RN74
139360	118990	129760	34120	45942	51125	E2QXY5
139110	188710	212120	227240	429150	463290	E2RSV9
138370	78634	89071	97107	188210	150490	F1PKR6
137430	117110	127740	132050	258150	220930	F1PDT7
137220	160780	187130	549800	1694300	1238500	F6Y2H4
136500	121030	142330	170520	335540	301500	E2QUY7
136410	95969	103590	116170	238530	191140	J9NWS6
136070	117120	130660	116860	298870	285300	F1P8K1
135760	128030	142260	138430	254310	233780	E2QUR2
135440	129100	159910	48345	88042	83358	J9P702
135090	137900	173590	675610	1512900	570420	F1PIA3
134030	96108	114060	62740	83404	69963	E2RJN6
133800	118360	142820	60198	98838	91010	E2RTL5
133620	108620	131380	63575	86684	82050	F1PAD5
133490	204020	222090	104570	480580	579300	REV__J9PAN1
130720	84605	102850	71386	213000	173740	E2QVG7
130300	224860	262450	50988	67359	79471	J9P9A6
129970	108070	124260	33884	68571	60206	E2RPI8
128960	148240	162620	110880	203570	208020	F1P797
128810	259750	244260	52129	41113	39622	J7I8M6
128760	93151	114060	80574	306260	272970	J9P798
128230	138430	160460	185450	305910	305630	E2RJ14
126750	134610	163080	25094	23660	22981	F1PLR3
126650	130180	151880	70211	105770	124950	E2RAU3
125850	124210	138700	216530	357320	355350	F1P624
125590	110500	117380	7828	6027	7235	A9RA72
125120	111390	123830	180960	381790	344510	E2RR33
124550	152230	73076	31048	17939	30374	F1PC33
123560	102080	105940	110630	111310	112740	F1PSR9
123060	176330	217630	638630	1302300	842060	CON__ENSEMBL:ENSBTAP00000016046
122250	132490	151510	130910	240600	212930	J9NX46
121740	83988	149150	64646	72691	58601	E2QSB5
121640	181470	221900	300680	1184600	1093000	F1Q1K6
121390	95159	98631	167280	256610	224780	E2R667
121160	207040	237060	688260	307090	316550	CON__Q3KUS7
120550	122030	114490	118010	200810	217750	E2R0S6
120010	103150	111460	77326	143000	132230	E2RSP4
119070	105620	118840	69677	127730	124450	O46605
118910	103860	127760	61340	79315	74330	F1PQT1
118500	292680	368350	53336	78307	74428	F1PHZ3
118150	126040	167840	37406	48928	41212	F1PG90
118110	125150	144710	58553	96593	108210	E2QS58
118110	113680	128670	26694	23901	22573	Q867A1
117250	119900	134750	67722	106390	98124	G3DTQ5
116890	113370	129800	170000	208380	162140	F6Y3U7
116880	100310	112530	45258	77090	71915	E2R8W7
115950	96788	114220	69034	131970	127070	F1Q260
115300	103300	101050	123020	211310	198680	E2RJ23
114700	113610	115960	162310	781920	827980	E2RLQ4
113590	110020	128960	74775	133970	127530	F1PJ65
113490	115060	131620	115800	434240	434010	E2RH09
112270	167590	194550	132860	318380	361790	E2RQ14
111720	59033	69878	29938	24667	29477	F1PW82
111400	143540	122740	108040	164980	199890	E2RST6
110910	118120	138600	231370	427710	365290	E2RQF8
110190	86918	52886	36363	81987	97061	F1PE38
110140	129290	169590	742580	887000	705890	F1PS66
109840	98404	117700	213530	502820	413110	J9NRH2
109780	91128	91862	88892	114300	117510	J9NRT0
109730	117630	130720	111250	81795	64300	E2RCI1
108030	75456	86701	32869	27294	30520	J9P5K5
106830	129750	140950	243360	392060	399640	F1Q1Q8
106660	101610	124270	172050	439490	392170	E2R9T6
106640	111190	123170	133520	270040	267310	F6XFY9
106180	85294	88656	7839.2	4491.5	4683.2	Q004A7
105610	108740	125700	100200	174790	195050	E2RMC1
105530	102200	106510	87198	229680	228790	J9P1N0
104190	106490	103700	129550	226740	223960	F6X4J2
103510	87837	92917	240560	526570	486670	E2QZ05
103370	69199	76084	54839	98686	71914	F1PM32
102670	69802	80963	57458	107630	80145	J9P897
102520	30339	45444	48399	59309	48200	CON__P48668
102420	154600	108990	49142	83905	80888	F1PVS7
102270	80504	96390	75823	113010	107000	F1PDI6
101320	87408	98410	97979	228620	204600	J9NWA6
100760	116140	123690	580950	332860	216940	CON__P00978
100740	79826	64464	32374	66002	67073	E2RK13
100330	132340	132230	101450	69237	82309	E2RJ30
100170	90382	104470	85857	129500	127980	F1PSZ2
99920	63456	83559	66197	125310	97749	E2R427
99741	137650	160950	62540	118130	94344	J9P0D8
99592	87335	98620	83919	318620	279750	E2RC86
99581	159820	171870	389130	219640	227050	CON__ENSEMBL:ENSBTAP00000038329
99352	115130	133330	181900	353830	362310	F1PYE3
98854	75976	84554	55547	65069	62476	E2QWP1
98520	135270	154080	116280	280250	269600	J9P357
98364	82768	91372	13939	14359	16764	J9NTM4
97850	104120	115040	254090	302060	323580	E2R2C3
97534	76510	70833	110360	202790	232810	E2RE57
96968	103070	113050	170370	419580	409390	E2RDL8
96933	90843	108040	41223	59314	56492	E2R4J1
96423	75163	59954	28112	44199	42358	C4TGH8
95363	120570	134400	74431	134690	169520	J9P5V4
94153	49895	62687	38236	41468	41717	F1PAR9
93315	108320	135880	23374	18877	19232	J9NU59
93137	94002	108090	207840	556530	472760	F1PBJ3
93007	91225	96604	127670	235250	218280	E2RPG9
91744	75684	92222	33687	66604	53166	J9P742
91560	81135	71242	197220	169290	157860	A0A3B0ITJ5
91459	87733	98593	117080	180090	173350	E2R2A4
91373	118730	137590	280470	221820	210260	CON__ENSEMBL:ENSBTAP00000018574
91277	92768	106200	53236	63751	62095	E2RE80
90954	233810	218670	102380	132320	117670	E2RQA6
90316	95642	105990	109580	213510	189500	E2RR97
90176	104480	110050	171660	324910	323810	F1PF02
89879	77604	90289	101430	325010	133370	F1P6Z2
89664	76385	92010	47838	76274	75604	J9NZ27
89200	77492	82195	53129	64931	62159	J9P6H6
88684	83233	83277	102150	200520	199320	E2QY43
87857	101580	111050	213110	216720	220830	F1PRE1
87461	83755	90184	70804	123320	115420	J9P2H0
87293	90381	100580	74292	114350	117780	B4YUE1
86716	71906	67480	114800	178190	193970	F1PB77
86306	57833	56760	42979	56625	60871	E2RHV3
86184	70290	80063	80894	163720	154880	E2R0K3
85589	89511	95227	139850	107180	111370	E2RNA7
85220	83597	88189	89745	124970	127530	F1PXT8
84835	92960	100260	35141	31622	31808	J9P095
84659	128620	53244	43982	31702	27553	REV__E2RT31
84073	91499	96508	117810	241130	273180	J9NT23
83789	50981	55737	57018	133890	120390	J9P4R8
83434	74740	74501	104160	321040	302200	J9P061
83261	77353	77738	137590	431300	380920	F1PFC6
83048	104100	149670	1395100	2690300	1908200	F2Z4Q7
82964	68555	81701	51416	76994	70591	E2QUA6
82963	82422	77706	26339	27169	32692	F1PGL0
82908	80089	96552	155030	292510	270970	F1PNP7
81970	90353	103870	74484	103950	106200	E2R516
81720	87594	103460	71722	216430	186150	F2Z4N1
81685	75465	85018	85315	168080	149780	E2RN00
81634	133520	173200	63631	101790	126340	F1PGJ2
81003	54196	77226	102970	135420	143670	F1P7E6
80821	100560	126350	154690	236650	260670	E2RM11
80759	104530	116950	75235	143350	138160	A0A1Y1FJ26
80613	62776	68559	108350	180750	155850	E2RF02
80395	63034	81176	29580	111910	104400	E2R885
80365	71818	86000	28953	30593	33829	F1P778
80226	78516	89535	52874	90998	82911	E2RRN8
79537	97304	97409	130960	99160	94370	REV__Q3HTU5
79220	66271	77635	44440	60806	58672	J9NYK7
79021	111540	119420	87920	157030	155570	E2RF42
78210	81264	113670	1016200	1528900	1368400	G1K2D8
78144	70260	74797	39547	45942	44194	F1P666
77869	96340	114360	252670	141310	141860	CON__P50448
77805	85800	99111	91508	183660	206300	F1PCG4
77769	80549	93730	107620	234130	234180	E2RGH5
77737	77629	94237	74972	130560	102910	J9NZU5
77621	62957	70665	45726	68476	59909	E2QW69
77505	123080	322550	266390	68908	87037	E2RSW0
77479	63087	81467	454700	102990	109440	F1PB11
76473	71500	81767	63064	72751	74456	E2RGI5
75751	71205	74175	87280	127800	129480	F1PVI2
75504	59951	68009	492850	190710	179840	F1PZL5
75086	73679	71607	71268	87788	78889	CON__P02662
74842	104360	124990	97450	35194	53451	REV__J9NX38
74456	73371	88924	50919	78761	77779	F1PL93
74169	66419	74254	88277	215510	201170	E2R2U3
73810	80760	95357	861020	381990	318420	E2RN38
73456	100240	107310	53440	81047	95685	F6Y478
73386	76447	94924	25485	39888	38405	F1P9W5
73147	86949	105750	269830	176090	167790	CON__Q0VCM5
73088	87740	109890	69849	147400	147070	E2R9G5
72950	95866	103270	292590	209140	193660	Q1ERT3
72873	59870	67999	31124	46181	41957	F1PCT1
72696	83454	99210	91215	246540	236190	F1PXG4
72682	85965	95527	211890	122500	122170	CON__Q9TT36
72675	186260	200560	146180	625870	599020	E2RBV8
71866	79284	80803	110760	122930	132240	S5THQ6
71450	68457	77461	77204	189230	177580	F1PPH7
71376	71891	77494	95343	190010	180730	F1PEK5
71315	73600	74225	94753	98206	112980	J9NU71
70455	58244	69746	35282	59713	57073	J9NY08
70053	70224	80532	85175	192000	169210	E2RI17
70003	48630	55653	12705	11231	11898	E2RFE1
70001	49659	48092	98328	222390	202240	E2R0S7
69990	67031	82394	150630	116010	110760	CON__ENSEMBL:ENSBTAP00000007350
69918	77788	88067	66975	121710	133340	E2RNQ8
69709	71598	82783	23808	22573	22159	F1PGX2
69692	64162	75832	57523	124490	123310	E2QXD1
69641	102240	129380	507960	1261500	943420	E2QW82
69271	68400	74539	93218	169930	154200	E2RE31
69249	64762	70816	52424	60851	59514	E2QWQ7
69222	65172	70166	21982	86453	37280	J9NW86
69169	68634	77395	32869	43518	41277	E2R5A5
68844	45123	52371	23533	67288	53365	E2R9G7
68714	34153	35335	15103	29615	26661	Q9XST9
68650	78255	91276	85382	117560	127270	E2R413
68503	73352	82413	101970	138710	135430	E2RF06
68416	65559	83916	31371	63072	59000	F1PS94
67994	98785	106730	382490	118710	160500	REV__F1PIU7
67767	57039	60591	77530	61535	56772	F1P9C6
67705	58061	66153	103200	142540	128170	F1PBX0
67054	63807	69005	52337	75319	89957	E2RB38
66695	78287	91131	73794	419900	322250	E2R068
66192	53793	53594	127820	295570	280160	E2RF39
65921	68540	77915	167130	184120	187540	K0J6C5
65545	56662	62203	49184	70662	70617	F1PQD3
65215	77861	98389	219270	146640	129320	CON__Q2KIS7
64806	64733	72261	50272	115180	112130	J9NVW6
64762	66385	72886	70600	139430	142040	E2RT63
64087	182730	176560	223190	126430	273890	REV__E2RN78
64034	65759	80249	49574	112560	102740	F1Q2M4
63610	63416	77526	35926	49788	53895	E2RF98
63524	50921	59406	76663	89170	85711	F1P9A7
63265	52584	59292	41842	106070	99040	J9P440
63246	97876	105980	35071	54765	58517	F1PGZ1
62360	82996	106130	272950	164480	132100	CON__Q95121
62316	86065	85246	54243	79726	88552	F6V4W0
61674	48554	53450	49128	117980	105700	J9P4C5
61603	76875	87555	222680	356500	315120	F1Q421
61578	86203	92136	51686	67913	70269	E2R7R0
61175	101960	109040	280920	140320	151990	F1PCZ0
60509	63978	69435	87914	210530	214330	J9PAP6
60390	53321	52640	60024	106120	106510	E2R6L1
60068	79271	100860	288830	384220	299790	J9P7U9
59757	55069	61839	86391	135550	134440	F1PAP9
59431	48818	57713	33838	52828	51807	E2QSE3
59148	38159	47187	11339	16943	13957	J9NXU1
59090	57869	62901	92259	146980	137580	E2R9K4
59047	50880	64583	486080	68724	67681	J9P6C2
58988	52351	64653	21508	28068	29428	E2QWD3
58600	50935	60624	41193	63893	61725	E2RMA1
58161	51124	52267	51200	82585	76257	F1PK29
57755	49760	58602	75421	115850	100060	J9PA14
57751	48976	57275	33025	53072	53045	E2QX84
56966	37590	46231	31209	84376	64311	J9PBD3
56542	68399	76712	251480	297350	268780	E2R4Q1
56315	44098	46131	35927	85818	75847	S5U7Q9
56122	34889	41616	72233	152470	149660	E2RRA3
55315	51358	61920	45388	89013	72494	A0A4D6CAB5
54473	49227	54885	88282	165900	158780	E2R1C3
53845	67397	82317	124220	271770	215950	J9NVY4
53779	81510	102270	19246	26361	25860	F1PH98
53124	54521	63171	87871	221010	245730	J9NZA0
52956	38236	43823	69142	125980	111940	F6UXI8
52701	57128	64288	51877	81494	81413	F1PYI3
52393	52912	63963	10016	13481	13038	J9NTG5
51894	49617	51933	62342	118020	107480	E2RH48
51859	38031	36794	186400	68017	45158	F1P8J6
51852	70283	69245	86276	129200	147130	E2RLF1
51824	55683	62402	42932	64010	57059	E2QYG5
51744	43163	52677	307440	82359	82922	REV__F1PQ17
51462	59339	72671	154130	89306	99711	E2RL01
51457	50143	60265	40803	73523	62355	E2R5H4
51415	45556	51162	37948	54859	52100	E2RG67
51274	59972	64560	56421	86706	97087	F1Q1R1
50913	67056	75240	60789	94645	93847	E2QV97
50479	64497	68513	183830	483170	693390	E2RAI4
49981	48401	53636	63754	75929	75076	J9P558
49812	46794	54226	47881	83151	83901	F1PQ10
49496	53165	61438	161710	348950	344350	F1Q3Y2
49368	52619	57043	71031	150800	136320	E2QTP2
49328	73693	95204	107670	426460	423060	F1PHT0
49216	62686	62219	50978	120110	112350	E2QTN0
48709	61660	75707	28276	44523	42335	F1PMM7
48693	63007	66343	88432	189040	191940	F1PRT4
48419	101230	104120	40552	48812	53449	E2R774
47745	174770	175080	71046	79657	87312	REV__L7N0K6
47640	53742	59488	36029	57959	59446	F6XST9
47627	63091	71759	139580	362460	310160	E2RHX5
47479	53076	66726	456360	401160	346720	E2QZN8
47368	64386	74931	186250	122990	118740	CON__Q3SZH5
47197	46552	52893	53096	82122	81141	F1PMC5
47180	31913	43330	41158	48998	44980	CON__P02533
47005	43117	49971	34414	51984	46107	E2RBC3
46971	64216	63911	64141	95458	108800	J9P755
46958	46604	54155	113210	357240	258100	E2RJL1
46774	39801	45716	30841	59278	43981	E2REA4
46638	31810	33834	24234	73064	57917	E2RPE5
46033	39617	40627	77719	117510	109810	E2QWE0
45714	37526	41418	26438	33882	31201	F1PDP5
45510	37711	45800	27967	52848	48679	E2RRR8
45457	69091	67926	67728	76493	96274	J9NT37
45326	54281	64760	40737	83656	97134	E2R5P5
45225	46626	53959	27531	26810	30859	E2QX93
45165	74767	56350	74461	87079	77547	F1Q1F6
44956	37536	45729	25374	32689	31345	E2R2A1
44948	68276	74379	255840	127300	115440	CON__REFSEQ:XP_585019
44441	52112	58278	51018	100770	93169	J9NUS5
44429	50393	50280	49372	119880	109080	F1PIR1
43985	44399	48251	31589	54907	55780	E2QVU9
43758	45488	50434	56026	115410	124560	E2QXR8
43219	39190	35995	31992	49029	48940	J9JHH0
43073	44209	47557	51559	87853	76384	E2R1R4
42988	42224	47956	34217	48151	44603	F1P9Z4
42188	39744	47608	43040	34558	34624	E2R071
41110	48124	58734	32948	56750	61322	J9P2G7
40917	38262	45744	55138	76066	69280	F1PYW3
40836	42584	46505	15622	17065	20380	E2RDJ9
40468	22163	26424	38611	92652	68228	J9PAS9
40036	50229	56538	104960	165180	161870	F1PGS1
39750	31840	34286	32030	35422	34833	J9P8P9
39726	30619	32759	36516	49140	43258	E2RNT3
39320	17793	20094	41823	67386	45665	A0A516UWM3
39312	35157	44941	26637	32730	27138	J9NVU0
38870	45721	51907	86513	263140	270170	J9NZ89
38360	26015	28090	22969	44656	41340	F6UKT8
38061	43976	49720	30377	51772	49878	F1P8U3
37887	46015	47599	50979	73196	67248	E2R949
37636	80377	89767	28507	50883	70245	F1P9Q3
37490	41058	49668	30770	49374	50055	E2R5J7
37416	37703	42612	63900	96229	94575	E2R4D5
37117	18255	20527	49298	87538	59682	E2R9I0
37020	24629	30479	70171	246680	183850	E2RDF9
36887	52954	54775	108850	99755	87612	CON__Q05B55
36312	192080	192010	10776	4927.1	5414.7	F1PP70
36015	35409	43448	298530	719280	353240	E2R755
35627	40231	49039	69093	94544	98577	F1PEC4
35329	44173	43732	27469	44211	45785	A0A077KFB1
35285	40490	50502	68983	240860	126360	E2RNL2
35171	43068	46465	43502	72461	60920	J9P1Q5
35138	21548	27541	52474	185400	139690	E2RRD2
34960	41303	45496	22928	28408	32893	F1PWQ1
34665	18740	21304	8922.5	29504	23375	A0A0A0MPD2
34591	27629	34978	11553	19913	17139	F1PEH9
34425	35076	37943	48023	38044	35573	CON__Q2KIT0
34273	35762	29384	20292	38584	43525	W8E199
34174	32049	34109	61076	124010	97481	F1Q067
33786	78340	82603	102640	145590	176590	E2RA21
33785	32716	35729	20352	40993	43312	J9P4S8
33479	37108	34863	29407	68804	75196	J9P7X9
33122	42299	48381	14492	11192	10965	F1PY18
33100	29672	32829	27555	46236	44954	J9NT20
32927	27139	30723	29920	60952	55878	E2QU31
32607	31408	35283	27328	44292	49832	F1PLU5
32599	29264	31390	26938	56296	51588	E2RL17
31865	27096	32504	37876	85319	87678	E2RMR3
31646	44856	51077	19826	32057	19660	F1PJM1
31617	25212	27482	40788	70133	62103	E2R7R8
30895	32335	36016	15290	21032	19339	E2RM30
30687	40131	43915	107830	78335	60586	CON__ENSEMBL:ENSBTAP00000014147
30380	29118	37775	96343	318130	271740	E2RR73
30357	23052	26345	22576	29706	27957	E2RPL0
30207	37484	40403	60700	80329	91630	F1PI70
30108	27712	31472	41643	115060	87641	J9P870
30028	33417	33854	139840	224910	212870	F1PN20
29763	27580	30841	35167	61078	59746	F1PB95
29277	40577	44100	15576	16702	18567	F1PMC8
29243	22254	22889	27753	67605	56337	E2R4Y6
29123	51350	59595	62981	33764	45682	E2RK19
28760	26660	32563	9951.5	24724	23236	J9P013
28528	25454	29114	21705	23001	24987	E2QX20
28425	20014	21611	22842	44081	37799	E2R7S5
28406	23964	30519	20933	32801	28396	E2R9R1
27929	24880	33777	12429	13514	10070	E2RFH1
27743	32581	36976	26514	44064	40735	E2RAI8
27706	23115	26892	29880	53179	49830	J9P7Y9
27599	34450	36911	25212	47369	50335	J9NT16
27397	22985	23999	26354	32526	30266	F6XWJ1
27251	28156	33499	53723	116020	100030	E2RH91
27044	36298	40479	32876	56238	49505	E2R2N2
26944	27210	28087	22044	28717	33739	J9NWR1
26633	15394	11290	27260	75809	48419	J9NYM0
26598	22957	25732	27240	37488	35627	J9P6K0
26424	30275	38350	32490	38859	40181	F1PIC7
26111	27754	34398	35980	95186	86692	E2RID8
26110	32197	41076	38346	61398	61717	J9P923
26007	20920	18269	42910	147230	139770	E2QXZ1
25932	17441	19954	11235	15367	14741	F1PUU5
25833	25088	29141	28863	42179	43006	E2R2V4
25786	27999	26223	38109	43999	46480	F1Q332
25709	28071	29603	35754	72277	69343	F6UVH0
25662	34238	42137	23845	43131	59754	Q4GX48
25588	13118	16005	10429	12489	9322.3	E2QUZ0
25499	31031	42744	61266	50309	53813	CON__Q32PJ2
25494	32314	41092	65618	121350	106230	F1PQU6
25360	30081	36465	23203	28973	30017	F1PGI1
25334	30928	38515	33179	88074	76007	J9P2I8
25209	27307	30503	27873	49426	48968	J9P388
24977	21321	25278	4335.2	2412.1	2805.1	Q6LAA1
24910	24945	30704	21176	26800	25897	E2RR96
24710	24387	25161	40615	55490	59512	E2RGK2
24663	25386	26027	11682	28324	27020	F1Q0U7
24499	30693	35295	86869	103930	103970	E2QZA8
24457	25640	30253	17232	36741	33719	E2RSK6
24310	15880	17833	17064	18596	15642	J9P9D2
24162	38023	45214	76580	63020	62747	CON__Q32PI4
24102	26119	27901	21189	27209	28921	L7N0B9
24021	30202	35128	25384	48593	45981	F6XQ49
23992	22319	26852	22150	26800	25966	E2RQE7
23975	30864	38096	38014	113140	105220	F1PU14
23923	35708	42515	23731	48652	51491	E2QWF5
23838	33012	37048	57680	68046	44101	F1PFM6
23812	23809	25401	1518.4	480.81	486.42	E2RAW5
23757	28117	31178	201700	350690	370020	F1P7H1
23668	20712	19086	22345	41502	39379	F1PFD6
23537	23684	30868	60975	79323	77388	F1PLE9
23391	21602	22273	19210	42607	38078	H9GWA6
23319	22530	26901	17273	25624	22683	E2RHZ1
23308	26404	31682	17393	24097	23831	F1P699
23203	30172	30522	16491	21280	22753	F1PA94
23057	17605	21117	26130	70802	61550	J9P098
22943	23013	26450	21957	34667	33293	F1PU09
22748	25485	23656	29134	59264	61167	J9NSL6
22236	17351	22078	20163	35393	31021	E2RAT8
22077	28569	33492	97022	60431	53383	CON__ENSEMBL:ENSBTAP00000033053
21951	32948	30626	30149	67867	93298	E2RAT6
21943	27087	29579	18994	39214	43224	E2RCL4
21899	26018	37166	68674	41971	36318	CON__P17690
21893	25865	30457	24904	30676	34425	Q3HTT4
21877	20253	20710	20860	29541	22892	L7N0H5
21676	19364	20647	16471	35609	25420	F1PQL8
21372	20142	20754	19307	36923	37134	F1PPR0
21248	18944	22104	28059	99062	90331	J9NRQ9
20780	15218	19266	4821.7	5286.2	4792.2	F1P625
20556	22862	25154	21431	22379	26775	E2RTM5
20503	14237	17546	21730	39616	34250	E2RPY9
20483	56313	60034	12651	23209	33774	F1P789
20354	19035	22102	27566	59855	41110	E2REK3
20284	21877	25542	9785.7	14128	13907	F1PA88
20127	16226	17978	13743	23929	22991	J9P9U3
19841	20838	23125	31470	50543	51470	F1Q3E7
19666	21011	25624	14849	27663	27694	J9P1V7
19438	14619	8175.9	3058.4	3930.6	5093.5	F1PM72
19412	16593	18134	19522	38611	35355	J9P615
19366	18073	20404	23498	30559	31829	F6UNY1
19301	17169	16484	13467	29147	33351	J9NVU2
19091	11556	14367	15875	22609	20394	E2RL38
19062	15641	17352	16720	34090	35567	F6UZV8
18937	33837	34435	21265	41938	64322	F1PQ43
18300	26064	29111	20309	49472	49767	E2QRT0
18180	24767	26165	36002	60418	62767	F1PL63
18077	22404	25023	41246	87162	85023	E2R7A3
17606	18896	19841	37101	90732	85645	E2RMA7
17588	19100	22339	20320	34191	31242	E2RRD1
17467	13821	17083	6852.5	9983.2	9269.9	J9NU50
17212	16429	18550	31118	64158	50853	F1PBW1
17200	14540	17253	19764	47541	45162	F6X571
17162	21266	21476	30696	65962	72192	REV__J9P5L3
17067	15019	18751	11901	13576	14397	F1PGB1
17067	16255	20931	11103	20606	19476	F1PQ68
16846	37083	40066	17085	24821	19741	J9NWD8
16795	14430	17249	25061	53976	55886	F1PMS4
16718	15208	17740	14416	18708	16744	J9P1Q9
16565	21564	26949	11590	24902	24414	J9NWY5
16550	14821	16271	20215	53981	45158	E2RCF9
16514	24663	24519	16157	41851	39714	E2QW49
16366	19987	20141	18470	31637	29584	E2QWE3
16320	13557	16056	16227	25811	23553	E2R273
16306	20447	24878	68637	98609	96406	E2RS79
16298	9892.6	11116	14965	16950	13033	CON__P02666
15863	14641	16089	13121	25143	22367	J9NXV2
15618	12574	15561	20157	26164	24816	J9P5H8
15429	14847	17274	28697	46022	42932	F1P9U4
15262	16380	18807	45962	105010	127520	A0A346JM02
15170	22759	35216	13193	2235.4	2501	Q95LE0
15156	15730	19556	143310	259610	175180	G1K2A7
15145	24665	27779	12038	17438	17450	E2R529
15011	28667	32089	42875	100270	153560	REV__J9NYT3
14921	11539	13216	5722.1	7775.9	8495.7	E2RRW6
14903	16615	16082	14984	45606	51538	F1PGI9
14849	16893	18824	21593	34543	29718	F6V8H1
14472	15227	17193	22625	24100	23565	J9NZX7
14455	21674	24784	23550	27774	29806	E2R7F2
14245	14807	14338	22995	65365	59365	F1PTE0
14170	18516	21195	17387	29480	27729	E2QYG6
13855	17532	19441	10511	17180	19818	E2RD86
13756	49301	43947	24722	21183	32628	REV__F1P8R2
13743	20731	25292	4901.5	4988.3	4487.8	F1PG57
13601	17999	20465	4803.3	7663.4	6556.8	K0J2W9
13524	12719	15281	20241	17715	17610	J9NZP4
13439	16866	17431	10534	10375	10945	J9P4N9
13194	14319	17924	36090	28973	25265	CON__ENSEMBL:ENSBTAP00000031360
13168	14928	12799	9205.4	37292	18216	J9PBI1
12800	15767	16122	13633	23864	22798	V5KXV2
12721	12548	12332	7225.2	13316	12621	F1PZ09
12672	12735	11841	28549	33570	36970	F1PHW5
12371	10043	7535.2	13981	32793	36458	E2REA8
12345	21610	25699	55996	36360	42605	Q9XSS9
12165	8812	4067.3	5943.5	11867	12785	E2RHY0
12133	16118	18844	16108	30100	28611	J9P8J3
12004	12082	14253	14375	21996	19881	E2QUV5
11562	9248.3	4638.9	6869.1	24795	20621	J9PAW9
11494	12149	13616	16920	17318	14993	CON__Q05443
11278	14878	14675	13563	16988	21575	F1PZA1
11233	12602	15609	11134	32816	30290	F1PRL0
11163	14219	16119	24272	21879	19057	CON__Q3MHN2
10964	10074	12570	15252	18552	16149	J9P4X0
10945	11798	13449	11061	11755	13378	F1P9K1
10927	9700.8	11316	15000	20966	18530	J9PAY5
10838	7275.9	9259.3	7399.1	8540.7	7658.2	J9P8T8
10530	8188	10324	9147.3	11625	11600	F6V659
10526	7380	4092.2	726.59	688.34	821.68	E2QUR0
10517	12640	13222	13717	17521	18410	F1P7P3
10424	7666.8	7907.5	16150	35738	28970	J9PB22
10388	12938	13936	19706	29597	30775	J9NX01
10347	10226	12443	33508	120500	82086	J9P8K2
10195	9329.4	11144	11686	20733	19772	F1Q1V0
9960.3	12945	16428	58074	150400	113510	F1PLV6
9848.4	11537	11278	17143	31267	28378	E2RL02
9804.6	10050	12667	5464.9	9311.8	10882	J9NYN4
9708	10411	13886	13251	55584	27748	Q5I4H7
9662.1	9925.9	10372	12295	22169	20102	F1PYA6
9555	14809	16579	19641	85162	80271	E2RJL3
9362	10892	12937	12068	16714	15883	F1PWG0
9304.6	11119	11509	22633	24472	33890	E2RH71
8962.1	7819.9	8046.9	6432.5	8268	7728.4	F1PCU2
8935.5	8749.6	9443.8	9533.1	14125	13807	Q38JA9
8721.4	7676.3	9747.3	10312	19027	15985	E2R1V2
8631.5	7438.2	8444.8	5741.8	6300.9	6107.1	Q6JDJ2
8597.2	46142	74968	26061	18340	34949	F1P7Q1
8544.1	11329	13293	10697	19994	20860	F1Q1P7
8511.5	8879.7	10382	12531	18100	17047	E2RHZ8
8358.2	12943	14745	3622.4	5926.8	7785.2	E2R2Z8
8056.5	6508.1	8073.4	6787.3	9400.8	9310.1	F1P732
7908.3	6569.7	6607.3	4385.4	8273.4	8498.2	E2RI60
7688.3	5568.4	3467.7	8462.5	26864	35951	F1PBK4
7655.4	6076.9	7656.1	9183.9	8506.1	7848.1	E2RKU5
7607.5	8485	9766.6	8530.3	10400	10916	F6V5M4
7531.1	8262.5	9579.3	3985.6	6387.4	6013	F6XLZ4
7357.3	7693.1	9210.8	5559.9	11300	9677.7	F6XD28
7301.4	7191.2	10156	21321	25050	20751	E2RF52
7241.2	7921.2	8285.2	8927.3	14240	14455	F1P9P5
7215.8	6675.5	7321.2	4105.7	8821.9	6280.1	F1P7F8
7014.8	6155.6	6589.1	6068.4	9195.2	8444.3	J9NVL3
6974.8	12531	14519	59552	20673	32681	REV__A0A3B0ITJ5
6952.8	7098.3	8542.7	13252	11338	11074	CON__ENSEMBL:ENSBTAP00000013050
6606	5889.2	7087.4	6154.4	8615	8807.3	F1P926
6496.6	8896.6	10423	33905	18483	15504	F6XTZ1
6458.9	7336.3	8842.2	9942.4	9855.5	9836.1	F1P7Y6
6446.9	6625.6	6968	5941.3	6898.7	8622.1	F1PVY2
6294.9	6787.2	8231.7	2254.4	2933.7	3712.3	J9NUD2
6206.3	7661.1	8655.8	5855.2	7609.2	7373	E2R020
5874.1	7479.8	8611.1	14861	25473	24235	E2R6Z2
5742.6	5253.3	6542.9	8486.3	50195	39248	E2R7P6
5694.6	6314.5	6664.7	7217.1	8594.2	9193.6	E2QZ13
5655.9	6032.1	4471.6	2340.7	4828.6	5439.5	I3RSH9
5532.6	11072	14680	7675.5	20222	19962	E2RAT5
5473	7360.6	8749.3	7649.6	10945	9650.2	E2RR85
5371	9085.4	10382	10469	80692	54310	E2RHX1
5027.5	4985	5541.7	7028.4	12660	11321	J9NZA2
4909.3	3663.3	4835.8	11456	10857	9374.6	F1P787
4900	4369	4694.2	5890	15497	16674	F6UMY5
4864.5	5168.9	2708.2	1677.9	3754.5	5216.1	E2R1D2
4859.3	5823.2	6982.7	4679	5899.5	6383.3	J9P2T7
4465.1	6829.1	9411	44264	65071	47224	F1PBX4
4377.4	3091.3	3268.9	2493.1	2448.5	2463	E2RNQ7
4132.4	1223.6	1558.6	6231.9	4390.8	2096.2	F6V9A6
4001.1	4919.8	5396.4	4737	5253.2	4925.5	E2RB90
3918.5	3731	4762.6	4183.3	5770.9	5803.5	E2R0F2
3892.9	5632.3	5727	6241.2	10369	11122	E2RH14
3745.1	4308.7	5231.2	3545.2	4058.3	3254.2	E2R8M4
3620.3	3801.1	4862.4	4065.8	4498.5	4788	E2RPP4
3418.9	2440.8	1667.9	2621.2	6518.1	6043.1	J9NWC1
3385.9	4008.7	5311.9	4389.8	5330	5484.9	E2RAC6
3299	3815.5	4286.3	7202.8	13350	12453	E2R311
3069.7	3190.8	3616.8	3466	5626.1	5267.4	E2RM61
3065.3	5230.5	2499.5	540.64	1043.4	1010.4	E2RE16
3058.4	3324.9	3572.3	4670.4	5180.7	5473.9	F1PZP6
2974	5541.4	6273.2	1872.5	3198.8	3453.5	F1PUS7
2971.6	2564.9	3928.4	5880.4	5045.5	5317	F1PZ86
2811.5	2167.7	1455.8	3161.5	11537	11417	E2R680
2745.1	3581.4	3662.9	3618.6	4762	5654.9	J9P9D9
2676.5	4635.1	6240.8	5530.5	19582	16048	E2R944
2651.1	2573.3	3198.9	4257.1	4067.7	3772.6	E2QXV3
2534.4	3172.3	2936.8	3230.8	6611.3	5942.2	F1PL65
2458.7	2805.3	3357.7	4070.1	15861	16852	F1PZ47
2430	2535.2	2770.5	3211.2	6470.3	4971.8	J9P4N2
2184.7	2565.6	2293.7	2078.6	3550.3	3635.8	J9P2E5
2044.8	2596.1	7749.2	164910	170570	136980	F1PXB0
1963.6	2334.4	2121.9	3352.3	6872.1	7157.5	F1PSL9
1551	1804.4	913.22	2251.4	9838.9	10430	F6XM35
1493.6	2648.2	3351.1	2948.2	16688	17576	Q6UQF9
1127.9	848.96	923.52	1398.2	2949.6	3468.5	J9PB98
1049.9	789.17	1106.7	1043.2	2561.5	2330.3	F1PF61
895.14	1074.7	783.13	232.5	534.31	321.93	E2RGQ1
888.54	1045.5	895.28	1369.6	1670.8	1877.3	F6UYN9
781.24	635.54	611.16	1492.8	1626.8	1369.5	J9P7X1
589.86	352.08	490.09	121.77	248.49	264.53	E2RAV0
581.45	278.52	522.56	849.25	1791.2	1546.2	E2R3U9
539.55	628.36	1067.2	8730.8	4013.8	3064.8	E2R8C6
458.14	701.32	557.2	715.6	1511.4	998.53	J9P3C1
94.581	166.45	139.94	654.82	14306	1830.8	F1P9U6
0	0	0	0	0	0	A0A1B4XK92
0	0	0	0	0	0	A4GT58
0	0	0	0	0	0	F1PA99
0	0	0	0	0	0	CON__ENSEMBL:ENSBTAP00000006074
0	0	0	0	0	0	CON__ENSEMBL:ENSBTAP00000011227
0	0	0	0	0	0	CON__ENSEMBL:ENSBTAP00000023402
0	0	0	0	0	0	CON__Q2KJ62
0	0	0	0	0	0	CON__P02663
0	0	0	0	0	0	CON__Q29RQ1
0	0	0	0	0	0	CON__Q2HJF0
0	0	0	0	0	0	CON__Q2KIF2
0	0	0	0	0	0	CON__Q2KIH2
0	0	0	0	0	0	E2QRX1
0	0	0	0	0	0	E2QTL3
0	0	0	0	0	0	E2QUH1
0	0	0	0	0	0	E2QUQ3
0	0	0	0	0	0	E2QUQ6
0	0	0	0	0	0	E2QUT9
0	0	0	0	0	0	E2QUY2
0	0	0	0	0	0	J9P4Q6
0	0	0	0	0	0	E2QWK8
0	0	0	0	0	0	E2QWS2
0	0	0	0	0	0	E2RH59
0	0	0	0	0	0	F1PT27
0	0	0	0	0	0	E2QXU5
0	0	0	0	0	0	E2QXV5
0	0	0	0	0	0	E2QY42
0	0	0	0	0	0	S5U7Q1
0	0	0	0	0	0	E2QYL0
0	0	0	0	0	0	E2QZV5
0	0	0	0	0	0	E2R0Z0
0	0	0	0	0	0	E2R141
0	0	0	0	0	0	E2R186
0	0	0	0	0	0	E2R231
0	0	0	0	0	0	E2R4C1
0	0	0	0	0	0	J9P2A6
0	0	0	0	0	0	E2R4L0
0	0	0	0	0	0	E2R587
0	0	0	0	0	0	E2R5F1
0	0	0	0	0	0	J9P8V7
0	0	0	0	0	0	Q95KP5
0	0	0	0	0	0	E2R887
0	0	0	0	0	0	E2R8E5
0	0	0	0	0	0	J9NVR7
0	0	0	0	0	0	E2R9N5
0	0	0	0	0	0	E2R9U8
0	0	0	0	0	0	E2RBC5
0	0	0	0	0	0	E2RBJ1
0	0	0	0	0	0	E2RBU1
0	0	0	0	0	0	E2RC92
0	0	0	0	0	0	E2RCG1
0	0	0	0	0	0	J9NZJ6
0	0	0	0	0	0	E2RCW6
0	0	0	0	0	0	E2RD37
0	0	0	0	0	0	E2RE36
0	0	0	0	0	0	E2RE39
0	0	0	0	0	0	E2REJ7
0	0	0	0	0	0	E2REU8
0	0	0	0	0	0	E2RF16
0	0	0	0	0	0	J9P858
0	0	0	0	0	0	E2RFM0
0	0	0	0	0	0	E2RG27
0	0	0	0	0	0	J9P227
0	0	0	0	0	0	E2RGH9
0	0	0	0	0	0	E2RGZ5
0	0	0	0	0	0	E2RHR7
0	0	0	0	0	0	E2RIA8
0	0	0	0	0	0	E2RIU2
0	0	0	0	0	0	E2RIW7
0	0	0	0	0	0	E2RJ12
0	0	0	0	0	0	J9P6K9
0	0	0	0	0	0	E2RJ60
0	0	0	0	0	0	E2RJB9
0	0	0	0	0	0	J9NRU2
0	0	0	0	0	0	E2RK29
0	0	0	0	0	0	E2RKJ6
0	0	0	0	0	0	E2RL65
0	0	0	0	0	0	E2RLB2
0	0	0	0	0	0	E2RLP1
0	0	0	0	0	0	E2RLY5
0	0	0	0	0	0	E2RMI1
0	0	0	0	0	0	E2RMK6
0	0	0	0	0	0	E2RN02
0	0	0	0	0	0	E2RN45
0	0	0	0	0	0	E2RNC5
0	0	0	0	0	0	J9JHA9
0	0	0	0	0	0	E2RPG3
0	0	0	0	0	0	E2RPS3
0	0	0	0	0	0	E2RQG8
0	0	0	0	0	0	F1PYH4
0	0	0	0	0	0	E2RRS7
0	0	0	0	0	0	E2RTI3
0	0	0	0	0	0	J9NWR5
0	0	0	0	0	0	F1P660
0	0	0	0	0	0	F1P6G0
0	0	0	0	0	0	F1P7A9
0	0	0	0	0	0	F1P7L9
0	0	0	0	0	0	F1P7M0
0	0	0	0	0	0	F1P7V6
0	0	0	0	0	0	J9NZ68
0	0	0	0	0	0	F1P8G0
0	0	0	0	0	0	F1P8J8
0	0	0	0	0	0	J9NUR9
0	0	0	0	0	0	J9NTM1
0	0	0	0	0	0	F1P9V1
0	0	0	0	0	0	F1P9Z6
0	0	0	0	0	0	F1PB37
0	0	0	0	0	0	F1PBJ4
0	0	0	0	0	0	F1PBM0
0	0	0	0	0	0	F1PC15
0	0	0	0	0	0	F1PCE2
0	0	0	0	0	0	F1PCH0
0	0	0	0	0	0	F1PCK9
0	0	0	0	0	0	F1PCX9
0	0	0	0	0	0	F1PD54
0	0	0	0	0	0	F1PDF1
0	0	0	0	0	0	F1PEF0
0	0	0	0	0	0	O77704
0	0	0	0	0	0	F1PF03
0	0	0	0	0	0	J9NRP8
0	0	0	0	0	0	F1PFN8
0	0	0	0	0	0	F1PFR2
0	0	0	0	0	0	F1PGD5
0	0	0	0	0	0	F1PGP3
0	0	0	0	0	0	F1PGY6
0	0	0	0	0	0	F1PHZ1
0	0	0	0	0	0	F1PI83
0	0	0	0	0	0	F1PK63
0	0	0	0	0	0	F1PKV2
0	0	0	0	0	0	J9P5A2
0	0	0	0	0	0	F1PMA1
0	0	0	0	0	0	J9P444
0	0	0	0	0	0	J9PAA8
0	0	0	0	0	0	F1PN76
0	0	0	0	0	0	F1PNS2
0	0	0	0	0	0	F1PP33
0	0	0	0	0	0	F1PPL5
0	0	0	0	0	0	F1PPR8
0	0	0	0	0	0	F1PQ46
0	0	0	0	0	0	F1PQ79
0	0	0	0	0	0	F1PRW0
0	0	0	0	0	0	J9P2E3
0	0	0	0	0	0	F1PSR7
0	0	0	0	0	0	F1PU61
0	0	0	0	0	0	M1VEJ1
0	0	0	0	0	0	F1PUE0
0	0	0	0	0	0	F1PUE2
0	0	0	0	0	0	F1PUL4
0	0	0	0	0	0	F1PUU4
0	0	0	0	0	0	F1PV60
0	0	0	0	0	0	F1PVB6
0	0	0	0	0	0	F1PVE5
0	0	0	0	0	0	F1PVP9
0	0	0	0	0	0	F1PWE3
0	0	0	0	0	0	F1PWE6
0	0	0	0	0	0	F1PY49
0	0	0	0	0	0	F1PZK6
0	0	0	0	0	0	F1PZL7
0	0	0	0	0	0	F1PZW8
0	0	0	0	0	0	F1Q0L5
0	0	0	0	0	0	F1Q1C5
0	0	0	0	0	0	F1Q237
0	0	0	0	0	0	F1Q284
0	0	0	0	0	0	F1Q2J2
0	0	0	0	0	0	F1Q2X2
0	0	0	0	0	0	F1Q385
0	0	0	0	0	0	F1Q4F5
0	0	0	0	0	0	F6UZY1
0	0	0	0	0	0	F6V7F8
0	0	0	0	0	0	F6V9G5
0	0	0	0	0	0	F6XBJ5
0	0	0	0	0	0	F6XGU5
0	0	0	0	0	0	F6XHA7
0	0	0	0	0	0	F6XQ20
0	0	0	0	0	0	F6XQD6
0	0	0	0	0	0	F6XRK3
0	0	0	0	0	0	F6XXQ6
0	0	0	0	0	0	J9P992
0	0	0	0	0	0	G1K296
0	0	0	0	0	0	Q9XT64
0	0	0	0	0	0	J9NTY9
0	0	0	0	0	0	H9GW87
0	0	0	0	0	0	H9GWE2
0	0	0	0	0	0	J9NRV6
0	0	0	0	0	0	J9NS50
0	0	0	0	0	0	J9NTI3
0	0	0	0	0	0	J9NU25
0	0	0	0	0	0	J9NUG1
0	0	0	0	0	0	J9NWQ3
0	0	0	0	0	0	J9NXR3
0	0	0	0	0	0	J9P492
0	0	0	0	0	0	J9P7W8
0	0	0	0	0	0	J9PA46
0	0	0	0	0	0	J9PAU4
0	0	0	0	0	0	Q2Q423

**Table 2:** CHMp enriched exosomal protein list

-Log(p-value)	Difference(CHMp/CHMm)	Gene name
3.825927762	4.232747396	VCAN
3.446658115	4.144786835	SERPINF1
2.855851168	4.076141993	COL1A2
2.364573136	3.860753377	COL1A1
4.010542696	3.840995789	BGN
3.01330113	3.472952525	RARRES2
3.46470961	3.431823413	CSF1
2.957979378	3.404488246	PPIB
2.730731501	3.381497065	COL5A2
2.584980508	3.119366964	MMP19
3.200554284	2.939285914	ANGPT1
3.992483184	2.853436152	PTX3
2.247380194	2.708808899	SERPINE2
1.999240186	2.703020096	CA9
2.340838841	2.562550863	VIM
3.586221933	2.529642105	COL15A1
2.279769264	2.508499781	CALU
2.29022807	2.498077393	FBN1
4.078013061	2.314013799	FSTL1
2.033073433	2.303468704	CCDC80
2.435262128	2.303056717	DPYSL3
1.532973002	2.284193675	NCL
1.783534213	2.279807091	HP1BP3
2.579635598	2.162349701	LOXL2
1.642646981	2.157704989	SF3A2
1.905211843	2.153563182	LGALS1
2.312220276	2.063093821	DCTN2
3.15621547	2.059827805	LDHA
2.898016023	2.038072268	QPCT
3.80319952	2.027355194	LRP1
1.658663759	2.019675891	SNRNP70
3.353548868	1.965273539	LTBP1
1.92614043	1.950872421	LMNA
2.058117674	1.931167285	PDCL3
1.793949072	1.926321665	PLOD2
2.784330443	1.869923274	FLNA
3.316970744	1.850252151	HTRA1
2.456524848	1.807861328	DCTN1
2.382420122	1.78372701	PLA2G7
2.557440061	1.765195847	SPARC
3.041392709	1.753854116	COL5A1
2.568047412	1.675702413	PLOD1
1.35656781	1.664530436	NPM1
2.912536394	1.648955027	HAPLN3
1.364246165	1.637510935	LOC474472;YBX1
1.394159141	1.620957057	IGFBP7
1.65013999	1.564975103	SF3A1
2.505482247	1.542140961	LDHB
1.914909424	1.533444405	SND1
2.039593579	1.507152557	PKM
1.884925665	1.493344625	TPI1
1.570956741	1.486865362	ILF2
4.348776385	1.473365784	PLOD3
3.535210432	1.468423843	GPI
1.953482022	1.450750351	ACTN1
1.868517468	1.446697871	ALDOA
2.153515994	1.438435872	ECM1
1.799269881	1.438388824	CTSB
2.258058226	1.428877513	UGP2
1.862710543	1.425952276	PSMB5
2.040322167	1.420176188	EHD2
2.072235175	1.414199829	ACTR1B
1.797192652	1.405944824	DYNC1H1
1.825208991	1.386330287	PSMA6
2.242046087	1.380494436	LGALS3
1.597058613	1.372160594	SF3B1
2.303290321	1.354293187	TSPAN9
2.316514596	1.345461528	Tg;TG
3.946996683	1.3450044	HEXB
3.258978315	1.344204585	AHCY
1.550451453	1.326200803	ARCN1
1.706817155	1.311513901	HSP27;HSPB1
1.708554258	1.311414083	COPA
1.408369909	1.29447333	COPZ1
2.345958928	1.280286153	DPYSL2
1.636177274	1.275075277	SERPINE1
2.200055363	1.262718836	PSMA3
1.680226216	1.261900584	SF3B3
1.926596316	1.259480158	PSMA7
1.985258345	1.253908793	QSOX1
1.841587306	1.246202469	PSMB2
2.334829633	1.229386012	PSMB3
1.331715302	1.227722804	EIF3A
1.853659617	1.223726273	ENO1
2.122573335	1.221523285	CAND1
1.822941849	1.217698415	PSMA5
1.892662003	1.214228312	PSMB4

**Table 3:** CHMm enriched exosomal protein list

-log(p-value)	Difference(CHMp/CHMm)	Gene name
4.506375702	-1.254926682	PRSS22
2.291151336	-1.260751724	LOC100855903;YWHAZ
3.16926308	-1.2680041	Oas2;OAS2
2.877796008	-1.272038142	4F2hc;SLC3A2
2.461284871	-1.33093071	EDIL3
2.651022336	-1.373197556	MARCKSL1
1.460425708	-1.434253693	CYR61
3.709946753	-1.4405454	GLTP
3.035761035	-1.45499293	LAMA5
2.146898533	-1.468008041	FERMT1
3.139041149	-1.487693787	PLAU
3.909790758	-1.501808802	ADAMTS1
2.974811694	-1.582276026	EPB41L1
3.562466945	-1.631938299	ITGA2
3.45317765	-1.648342133	ARRDC1
3.689625424	-1.651777903	LAMB1
3.988619856	-1.697559357	LAMC1
4.478561433	-1.705554326	DSG2
2.189894031	-1.814268112	PLAT
3.321172408	-1.890741348	L1CAM
3.643548691	-1.915160497	CTNNA1
3.591474729	-1.995358149	ATP1B1
3.422291835	-2.020580928	TGFBI
3.893879143	-2.051908493	TIMP2
2.837414475	-2.061990738	FLT1
4.059538472	-2.062202454	GOLM1
4.989410104	-2.065231959	LAMA3
2.606813109	-2.079588572	THBS1
4.056597069	-2.08832105	ZDHHC5
3.406738656	-2.101538976	SLC44A2
4.862737461	-2.118820826	SEMA3C
3.732651284	-2.129861196	LSR
3.834112047	-2.182456334	CPD
2.498679744	-2.196196238	timp3;TIMP-3
3.754024745	-2.218083064	KIAA0040
4.164748799	-2.242208799	PROM2
3.829128818	-2.289141973	
4.784021165	-2.292790095	CXADR
3.929900312	-2.355760574	LAMB3
4.371178247	-2.359786034	VTCN1
3.515384416	-2.375387192	KRT19
2.964369917	-2.413296064	SDC1
4.487920622	-2.41403389	LAMC2
3.715175607	-2.446184476	IL1RAP
4.603869955	-2.557299614	GPRC5C
3.797982634	-2.609004974	PTGFRN
5.100710449	-2.623574257	ST14
3.400650117	-2.652560552	KRT7
5.324739264	-2.747966766	MMRN2
5.740500316	-2.793313344	GPRC5A
4.427999633	-2.828067144	AGRN
4.787621977	-2.836886724	SLC2A9
5.590810378	-2.971192042	TF
5.628379284	-3.04624176	FAM3D
3.946455631	-3.132736206	TINAGL1
2.767503537	-3.19803683	CLDN7
4.602169944	-3.2772096	INHBA
5.814537685	-3.343172073	TMPRSS11E
5.40084921	-3.421566645	S100A16
3.665249927	-3.558298111	EPCAM
4.417990435	-3.645421346	CDH1
4.347089343	-3.653319677	FGFBP1
4.221828618	-4.036827723	TMPRSS11B
3.505575643	-4.145844777	COL12A1
3.74733543	-4.169596354	S100A14

**Table 4:** Differentially expressed top 10 proteins in CHMp and CHMm-derived exosome

-Log10(p-value)	Differene (log2 CHMp/CHMm)	Gene name	Report intensity					
			CHMp_1	CHMp_2	CHMp_3	CHMm_1	CHMm_2	CHMm_3
3.825927762	4.232747396	VCAN	22.4696102	23.130125	22.8115883	18.2517414	18.4355888	19.0257511
3.446658115	4.144786835	SERPINF1	20.4119377	21.3593349	20.863781	16.3416576	16.6676102	17.1914253
2.855851168	4.076141993	COL1A2	22.7126141	21.9729519	21.4228573	17.4363213	17.8027821	18.6408939
2.364573136	3.860753377	COL1A1	23.5662785	22.3222084	21.7536869	18.0861778	18.5545368	19.419199
4.010542696	3.840995789	BGN	19.9547539	20.5440636	20.384058	16.2550659	16.3103294	16.7944927
3.01330113	3.472952525	RARRES2	18.1875172	19.4561939	18.4302883	15.1363106	15.1118288	15.4070024
3.46470961	3.431823413	CSF1	17.621851	18.419836	18.4972439	14.5360651	14.7791548	14.9282408
2.957979378	3.404488246	PPIB	21.0832329	22.3854809	21.5912189	18.1218681	18.1542244	18.5703754
2.730731501	3.381497065	COL5A2	23.3597107	22.7222366	22.1970959	18.8724041	19.2987614	19.9633865
2.584980508	3.119366964	MMP19	18.9543552	20.2667084	19.8475399	16.0876484	16.6415997	16.9812546
2.767503537	-3.19803683	CLDN7	16.8106461	16.8545284	16.4988022	20.686285	19.7997971	19.2720051
4.602169944	-3.2772096	INHBA	18.7562962	18.5668488	18.6019745	22.1732864	21.7049751	21.8784866
5.814537685	-3.343172073	TMPRSS11E	17.8801098	17.732975	17.9181767	21.2247028	21.088419	21.2476559
5.40084921	-3.421566645	S100A16	16.0853691	15.9640923	15.9379711	19.5739841	19.2752151	19.4029331
3.665249927	-3.558298111	EPCAM	18.4208241	17.7122993	17.5997238	21.6120281	21.2593136	21.5363998
4.417990435	-3.645421346	CDH1	18.0086479	17.600668	17.6528454	21.2072315	21.3393021	21.6518917
4.347089343	-3.653319677	FGFBP1	17.6992741	17.1020584	17.217598	20.8836784	21.0111351	21.0840759
4.221828618	-4.036827723	TMPRSS11B	18.6092434	18.0568485	18.0198193	22.4973125	22.0662174	22.2328644
3.505575643	-4.145844777	COL12A1	22.7758141	21.8359566	21.7053547	26.0335541	26.2941608	26.4269447
3.74733543	-4.169596354	S100A14	16.9048939	15.9426975	16.095644	20.5289803	20.3158398	20.6072044

### Protein interactions identified in CHMp and CHMm exosomal proteins

**Fig. 3:**
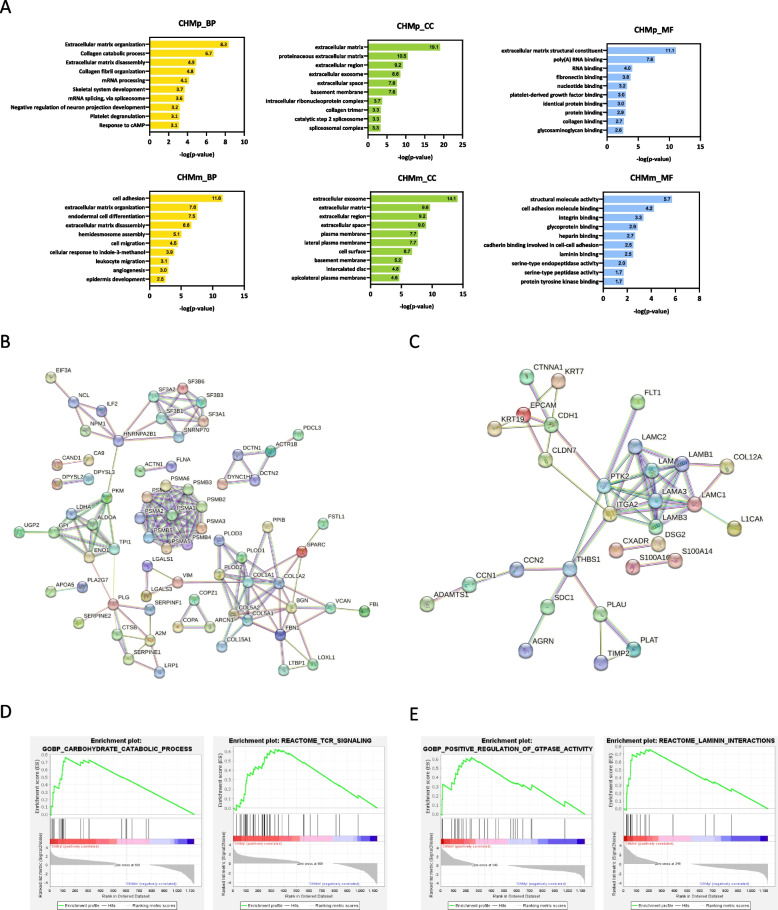
Comparative proteomic analysis between CHMp and CHMm exosomal proteins. **A** Gene Ontology (GO) analysis of statistically enriched in CHMp and CHMm exosomal proteins by the web tool DAVID v 6.8. (Yellow bars; BP, biological process. Green bars; CC, cellular component, blue bars; MF, molecular function). **B** STRING network analysis of differentially expressed proteins (DEPs) in CHMp exosomal proteins. Protein–Protein Interaction (PPI) network showing that CHMP exosomal proteins are enriched in proteasome, glycolysis/gluconeogenesis, splicing factor and extracellular matrix-collagen.** C** STRING network analysis of DEPs in CHMm exosomal proteins. CHMm exosomal proteins are enriched in Laminin. Proteins are shown as nodes. **D-E** Gene set enrichment analysis (GSEA) between DEPs in CHMp and CHMm exosomal proteins. **D** GSEA revealed carbohydrate catabolic process were significantly enriched in CHMp exosomal proteins. **E** Laminin pathways enriched in CHMm exosomal proteins

To gain a better understanding of the function of the identified exosomal proteins, we conducted Gene ontology (GO), STRING (Search Tool for the Retrieval of Interacting Genes) and Gene Set Enrichment Analysis (GSEA) (Fig. 3). GO analysis was conducted for each group, including biological process (BP), cellular component (CC), and molecular function (MF) (Fig. 3A). CHMp exosomal proteins were significantly enriched in Collagen, Poly (A) RNA, and protein bindings, whereas CHMm exosomal proteins were mainly involved in the extracellular matrix proteins organization and binding (Integrin, Laminin, and Cadherin). The protein interaction hubs of the CHMp and CHMm exosomal proteins were found to be completely different. The terms of "Proteasome", "Glycolysis/Gluconeogenesis", “Splicing factor” and "Extracellular matrix-collagen" were exclusively composed of CHMp exosomal proteins (Fig. 3B). On the other hand, interactions with laminin (LAMA3, LAMA5, LAMB1, LAMB3, LAMC1, and LAMC2) proteins were observed in CHMm exosomal proteins (Fig. 3C). These protein–protein interaction (PPI) hubs were also reflected in the Gene Set Enrichment Analysis (GSEA). The GO analysis of CHMp exosomal proteins revealed enrichment in the "carbohydrate catabolic process", while the Reactome analysis showed enrichment in "TCR signaling" (Fig. 3C). In addition, the GO analysis of CHMm exosomal proteins showed enrichment in the "positive regulation of GTPase activity", and the Reactome analysis indicated enrichment in "laminin interaction" (Fig. 3D). These results indicate that the composition of exosomal proteins differs between the two cell types, leading to distinct predicted functions. This highlights the potential functional variations of these exosomes in relation to their tumor microenvironment and target cells.

### Glycolysis enzymes were enriched in primary tumor derived exosomes

**Fig. 4:**
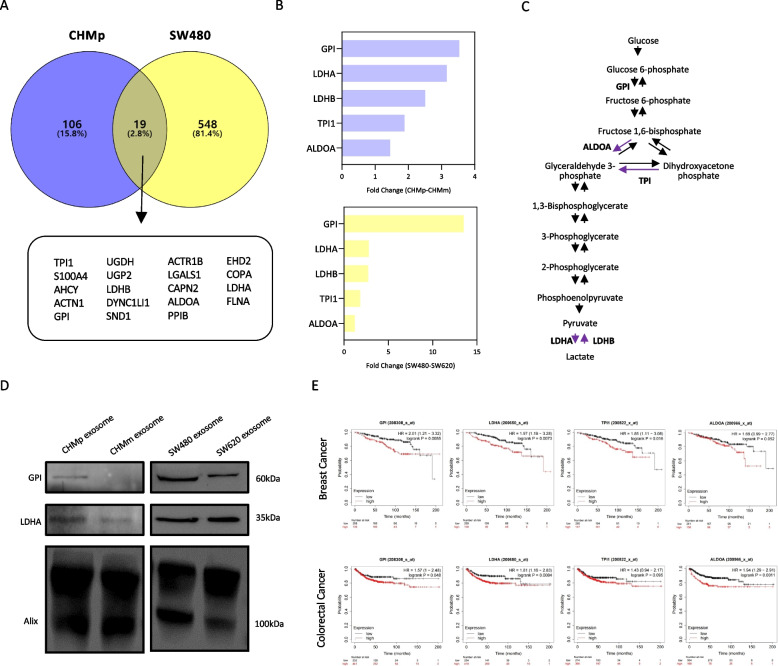
Comparative analysis of primary tumors-derived exosomal proteins between CHMp and SW480.** A** Venn diagram illustrating the 19 common proteins between CHMp and SW480 DEPs. Venn diagram visualized using the Venny 2.1. DEPs satisfying the fold change > 1.2 and p-value < 0.05. **B** The five proteins commonly enriched in both CHMp and SW480 exosomal proteins. (Purple bars; CHMp enriched, yellow bars; SW480 enriched). GPI showed highly enriched in both CHMp and SW480.** C** Glycolysis and gluconeogenesis pathway. Primary tumor enriched enzymes (GPI, LDHA, LDHB, TPI1, and ALDOA) were bolded.** D** Western blot analysis of primary tumor and metastases-derived exosomes for GPI and LDHA. GPI showed enrichment both in CHMp and SW480, whereas LDHA showed enrichment only in CHMp exosomes. Alix was used as loading control. **E** Kaplan–Meier survival curves of GPI, LDHA, TPI1, and ALDOA of breast cancer and colorectal cancer patients. High-level expressions of GPI, LDHA, TPI1 and ALDOA in breast and colorectal cancer patients showed worse overall survival outcomes. Kaplan–Meier plots derived from http://kmplot.com/analysis/

To investigate whether the differences observed in canine mammary gland tumor-derived exosomal proteins are applicable to other species and cancers, we conducted a comparative analysis using proteomic data from human primary (SW480) and metastatic (SW620) colorectal cancer-derived exosomes [27]. We selected SW480 enriched exosomal proteins with fold change (fold change) > 1.2 and *p*-value < 0.05 and compared them with CHMp exosomal proteins. Among the selected proteins, we found 19 proteins that were more abundant in both SW480 and CHMp exosomal proteins, with several proteins related to glycolysis/gluconeogenesis (GPI, LDHA, LDHB, TPI1, and ALDOA) being commonly enriched (Fig. 4A). Notably, GPI showed more than a three-fold enrichment in both the CHMp and SW480 primary tumor exosomes compared their respective metastases (Fig. 4B and Supporting Information Table 1). The glycolysis enzymes enriched in the exosomes of primary tumors are all involved in lactate production during the glycolysis process (Fig. 4C). Western blot showed that GPI proteins were significantly enriched in primary tumor-derived exosomes across species and cancers. LDHA proteins were enriched only in CHMp-derived exosomes (Fig. 4D). We confirmed the levels of Alix in exosomes to serve as a loading control. Among the genes of the five proteins selected, GPI, LDHA, TPI1, and ALDOA showed high expression in both breast cancer and colorectal cancer and were associated with poor patient prognosis (Fig. 4E). In summary, we identified glycolysis enzymes specifically enriched in the exosomes derived from primary tumors. These findings suggest that primary tumor-derived exosomes are likely to affect on the lactate production of neighboring tumor microenvironment or distant target cells and have implications for cancer prognosis.

## Discussion

Metastases exhibit unique characteristics and high heterogeneity due to the differential tumor microenvironment (TME) in which they reside [3, 28]. To gain a deeper understanding of the heterogeneity between primary tumors and metastases, we focused on analyzing proteins respectively. In our study, we employed LC–MS/MS to screen exosomal proteins, providing insights into the molecular composition of exosomes derived from primary tumors and metastases. Notably, our analysis revealed a significant enrichment of glycolysis enzymes specifically in the exosomes derived from primary tumors. This finding suggests that primary tumor-derived exosomes may play a role in modulating the metabolic landscape of the tumor microenvironment.

Canine mammary tumors (CMT) are the most common disease in female dogs. Recently, the use of CMT as a model for human breast cancer has been recognized in the field of comparative medicine. This approach is justified by the genetic and epigenetic proximity between dogs and humans, surpassing that of mice. Furthermore, human breast cancer and CMT not only share the commonality of being spontaneous cancers but also exhibit similarities in epidemiological, environmental, and pathological features, including histological and molecular heterogeneity.

We conducted a comparative analysis using the exosome proteomics data from human primary and metastatic colorectal cancer cells (SW480 and SW620). This analysis also revealed a significant enrichment of glycolysis enzymes in primary tumor-derived exosomes, consistent with our findings in CHMp exosomes. Glycolysis, a key metabolic pathway involved in energy production and biosynthesis in cancer cells [24], is closely associated with tumor metastasis [25]. Exosome-mediated metabolic reprogramming plays a crucial role in tumor microenvironment formation and tumor progression [26], affecting various surrounding cell types, including normal fibroblasts, cancer-associated fibroblasts (CAFs), mesenchymal cells (MSCs), endothelial cells (ECs), and immune cells. The key mediators involved in this process are the miRNAs and proteins within the exosomes. Exosomal miRNAs such as miR-105, miR-155, and miR-210 derived from cancer cells have been shown to increase glycolysis in CAFs, leading to enhanced lactate production that fuels cancer cell growth. Among exosomal proteins, VEGF found in exosomes stimulates glycolysis in endothelial cells, while the glycolytic enzyme PKM2 enhances glycolysis in MSCs [26].

A recent report revealed that exosomes derived from cancer cell lines with high metastatic potential contain a greater abundance of glycolytic enzymes [29]. Notably, GPI showed substantial enrichment in both primary tumor-derived exosomes and primary colorectal cancer-derived exosomes compared to their respective metastatic counterparts. In various cancers, GPI expression is increased by c-Myc and HIF-1 [30]. GPI knock-out (KO) has been shown to inhibit cancer cell growth [31], suggesting a significant role for GPI in cancer progression. Understanding the mechanisms underlying exosomal transfer of GPI and its impact on the TME indicates the importance of communication between cancer cells and their microenvironment. Further research is warranted to elucidate the specific role of exosomal GPI in mediating cancer cell growth and its implications for tumor progression and therapeutic interventions targeting the TME.

Not only GPI but also these glycolysis enzymes, except for LDHB, have been associated with patient’s poor prognosis in breast cancer and colorectal cancer. These findings suggest that primary tumor-derived exosomes may influence lactate production in the tumor microenvironment or distant cells, thereby impacting cancer prognosis [32, 33]. Our findings also indicate that lactate production enzymes enriched in the exosomes are not limited to a specific species or type of cancer, but represent a characteristic of primary tumors independent of species or cancer types. Moreover, exploring the enrichment of glycolysis enzymes in the exosomes may uncover their potential role in shaping the metabolic microenvironment.

Similar to glycolysis enzymes, splicing factors specifically enriched in primary tumors compared to metastases have been reported to play a role in the Epithelial-Mesenchymal Transition (EMT) during the metastatic progression of cancer [34]. This suggests their significant involvement in tumor progression. Splicing factors play a crucial role in RNA splicing, a process that transforms the initial RNA transcript (pre-mRNA) generated by the transcriptional apparatus into mature mRNA. Recently, it has been revealed that splicing factors are involved in the regulation of the Epithelial-Mesenchymal Transition (EMT) in the metastatic cascade of cancer. Moreover, numerous core splicing complexes (e.g., SF3B1, SF3B2, and SFRS1) in oncogenic Madin-Darby canine kidney cell-derived exosomes have been identified to promote metastatic progression [35]. Additionally, it has been reported that splicing components within exosomes are involved in the selective enrichment of miRNA. Splicing factors (SRSF1, EIF3B, TIA1) are implicated in the enrichment of pancreatic cancer-derived exosomal miRNA, particularly contributing to the exosome shuttling of miR-1246 [36]. Thus, exosomal spliceosome components, by participating in the selective shuttling of exosomal miRNA, imply that the functions manifested in cells may vary depending on which miRNA is shuttled into exosomes by spliceosome components. Exosomal miRNA, plays a crucial role in the tumor microenvironment. Considering the imbalanced enrichment of spliceosome components, further study is needed to investigate the differences in miRNA within exosomes derived from primary tumors and metastases.

The role of the proteasome in cancer involves its crucial function in maintaining proteostasis within cells by removing short-lived regulatory proteins and damaged proteins. The eukaryotic 26S proteasome is composed of the 20S core particle proteasome and the 19S regulatory particle (RP). The 20S core protein consists of 7 alpha subunits and 7 beta subunits, with the alpha subunits forming a cylindrical structure. Notably, beta subunits, specifically beta 1, beta 2, and beta 5, possess hydrolytic activity, cleaving the C-terminal peptide bond behind specific amino acids to exhibit Thr protease activity [37]. Exosomes derived from primary cancer contain a higher abundance of proteasome subunits compared to those from metastatic cancer. Specifically, they significantly contain all alpha subunits except for alpha subunit 4, and beta subunits with catalytic activity, such as beta subunit 2, 5, 3, and 4. Recent proteomic analyses of exosomal proteins have revealed the presence of numerous proteasome subunits in exosomes [38]. In exosomes derived from a mouse model of prostate cancer, all subunits of the 20S proteasome were confirmed, and tumor-associated macrophages exhibited a higher abundance of proteasomes in exosomes compared to naïve macrophages [38]. Moreover, studies suggest that exosomes can induce angiogenesis and enhance metastatic activity. Additionally, in mice with vascular injury, an increase in apoptotic exosomes containing the 20S proteasome core has been reported, indicating potential implications in autoantibody production and rejection acceleration [39]. Although the presence of proteasomes in exosomes has been observed, their exact role within exosomes remains elusive. Whether exosomes serve to deliver proteasomes to other cells or discard intracellular proteasomes is an area yet to be fully understood. Further research is needed to unravel the specific purpose, origin, mechanisms, and substrate specificity of these 20S proteasomes for understanding the role of primary tumor-derived exosomes. In this study, we report the identification of proteasome subunits are unique to the CHMp exosomal protein.

Overall, unraveling the connections between primary tumors and glycolysis-related exosomal proteins can provide a deeper understanding of the mechanisms driving cancer progression and open new avenues for the development of targeted therapeutic strategies aimed at disrupting these processes.

## Conclusions

In this study, through LC–MS/MS, we discovered significant differences between the CHMp and CHMm-derived exosomal proteins. Enrichment of glycolysis enzymes (GPI, LDHA, LDHB, TPI1, and ALDOA) in exosomes derived from primary tumors indicates their potential contribution to metabolic reprogramming on nearby or distant target cells. However, this study is limited by the use of only two cell lines, and the specific mechanisms by which enzymes involved in this process may impact tumor microenvironment cells have not been elucidated. In future studies, we will further expand our analysis by including different types of primary and metastatic cell lines for comparison and investigate the specific functions of these glycolysis enzymes while exploring their roles in tumorigenesis. In summary, larger and more in-depth studies are crucially needed to elucidate the roles of glycolysis enzymes derived from primary tumors.

### Supplementary Information

**Additional file 1. **

**Additional file 2. **

## Data Availability

All data supporting the findings in this study can be obtained from the corresponding author upon reasonable request.

## Abbreviations

LC–MS/MS: Liquid chromatography—mass spectrometry
GPI: Glucose phosphate isomerase
LDHA: Lactate dehydrogenase A
LDHB: Lactate dehydrogenase B
TPI1: Triosephosphate Isomerase 1
ALDOA: Aldolase A
TME: Tumor microenvironment
FASP: Filter-aided sample preparation
SDB-RPS: Poly(styrenedivinylbenzene) reverse phase sulfonate
TEM: Transmission electron microscope
DLS: Dynamic light scattering
GAPDH: Glyceraldehyde-3-phosphate dehydrogenase
HSP90B1: Heat Shock Protein 90 Beta Family Member 1
HIST2H3A: Histone cluster 2 H3 family
SUB1: SUB1 Regulator of Transcription
MAN2A1: Mannosidase Alpha Class 2A Member 1
BTGALT1: β-1, 4-Galactosyltransferase gene
LAMP1: Lysosomal Associated Membrane Protein 1
ITGB5: Integrin Subunit Beta
PPI: Protein-protein interaction
STRING: Search Tool for the Retrieval of Interacting Genes
GSEA: Gene Set Enrichment Analysis
