# Supplementary figures and images for: Exosome proteomes reveal glycolysis-related enzyme enrichment in primary canine mammary gland tumor compared to metastases
Source: Proteome Sci. 2024 Feb 28;22:4. doi: 10.1186/s12953-023-00226-5 (PMC10900604; doi:10.1186/s12953-023-00226-5)

A

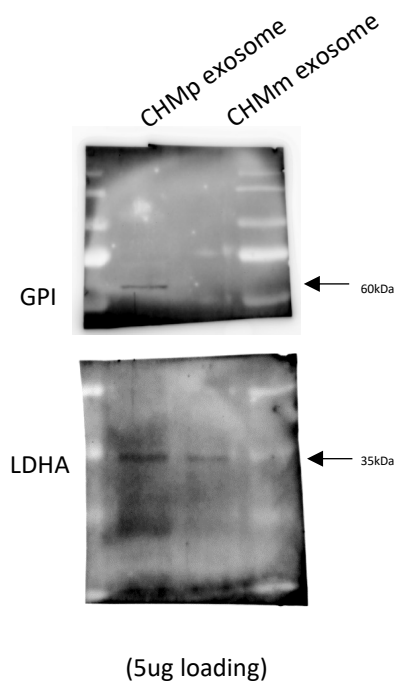

B

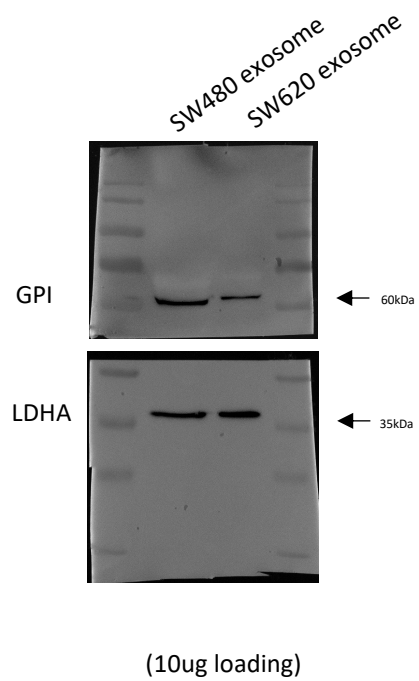

C

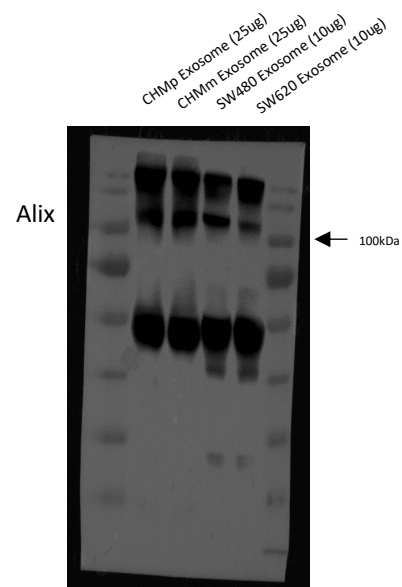

Supplement: Supplementary file 1 — Additional file 1. [file 12953_2023_226_MOESM1_ESM.pdf]
